# The effects of whole-body vibration therapy on immune and brain functioning: current insights in the underlying cellular and molecular mechanisms

**DOI:** 10.3389/fneur.2024.1422152

**Published:** 2024-07-31

**Authors:** Gargi Ahuja, Y. Laurisa Arenales Arauz, Marieke J. G. van Heuvelen, Arjan Kortholt, Tamás Oroszi, Eddy A. van der Zee

**Affiliations:** ^1^Molecular Neurobiology, Groningen Institute for Evolutionary Life Sciences (GELIFES), University of Groningen, Groningen, Netherlands; ^2^Department of Cell Biochemistry, University of Groningen, Groningen, Netherlands; ^3^Department of Human Movement Sciences, University Medical Center Groningen, University of Groningen, Groningen, Netherlands; ^4^Human Physiology and Sports Physiotherapy Research Group, Faculty of Physical Education and Physiotherapy, Vrije Universiteit Brussel, Brussel, Belgium

**Keywords:** passive exercise, neuroinflammation, neurotransmission, neuroprotection, neurodegeneration, vibration therapy

## Abstract

Whole-body vibration (WBV) therapy is a way of passive exercise in which subjects are exposed to mild and well-controlled mechanical vibrations through a vibrating platform. For a long time, studies have focused on the effects and applications of WBV to enhance musculoskeletal performance in athletes and patients suffering from musculoskeletal disorders. Recent evidence points toward the positive effect of WBV on the brain and its therapeutic potential in brain disorders. Research being done in the field gradually reveals cellular and molecular mechanisms underlying WBV affecting the body and brain. Particularly, the influence of WBV on immune and brain function is a growing field that warrants an up-to-date and integrated review. Immune function is closely intertwined with brain functioning and plays a significant role in various brain disorders. Dysregulation of the immune response is linked to conditions such as neuroinflammation, neurodegenerative diseases, and mood disorders, highlighting the crucial connection between the immune system and the brain. This review aims to explore the impact of WBV on the cellular and molecular pathways involved in immune and brain functions. Understanding the effects of WBV at a cellular and molecular level will aid in optimizing WBV protocols to improve its therapeutic potential for brain disorders.

## 1 Introduction

WBV therapy involves exposing individuals to mechanical vibrations through a specialized platform (1). WBV exploits an organism’s vibrational sense, a widespread phenomenon in the animal kingdom (2–4). This innate capacity to detect vibrations is believed to have evolutionary significance, enhancing an organism’s connection with its environment (5, 6). Humans are susceptible to mechanical stimulations (often encountered in an oscillatory form), ranging in frequency from ~1 Hz up to at least 100 kHz (7).

Mechanoreceptors in human skin process these vibrations, relaying signals to the brain through the spinal cord. In addition to those in the skin, Piezo1 and Piezo2 proteins are mechanosensitive ion channels located in the internal tissues of mammals. These channels trigger mechanotransduction, leading to the production of various hormonal and non-hormonal signaling molecules that affect cellular and metabolic physiology (8). This relaying influences physiological processes across the whole body.

While high-frequency (>100 Hz) and high-intensity (>10 mm) vibrations can lead to musculoskeletal impairment, and increase the risk of developing hand-arm vibration syndrome (9, 10), low-frequency (10–50 Hz) and low-amplitude (<10 mm) vibrations have demonstrated positive effects, particularly in therapeutic applications (3).

WBV can be viewed as a form of passive exercise. In a nutshell: an active form of exercise is where a person exerts force and puts in the effort to complete a move, whereas, in passive exercise, minimal movement is required by the individual. Either someone else moves the body for them or some machines induce exercise-like effects which is what happens in the case of WBV using vibrating platforms (3). WBV, to a level, helps to achieve the beneficial effects of conventional exercises. In older adults, WBV may improve muscle strength, power, and balance compared to untrained individuals (11–13). In many cases, WBV is used as an additional regime to the basic exercising routines as it has been shown to augment the effects of resistance training and other forms of physical exercises. It has been demonstrated that WBV exerts a positive effect on upper-limb performance in combination with exercise (14). Evidence also suggests that WBV may be a good alternative to stretching as a warm-up as it boosts strength exercises in the older population and also seems to enhance cyclist performance (15, 16). Furthermore, WBV may be a useful co-adjuvant in conventional rehabilitation therapy to improve postural stability and achieve better physical, functional, and emotional outcomes in individuals undergoing vestibular rehabilitation (17). This is further strengthened by the fact that WBV stands out as a convenient and highly accessible exercise option. WBV requires less effort, is time efficient, cost-effective, and suitable for various settings and, therefore, adaptable for home or clinical use. Particularly for individuals facing physical limitations or lacking motivation due to factors like frailty, depression, or other mental health challenges, WBV emerges as an accessible and effective means to initiate and enhance physical activity (18, 19).

While the primary focus of studies has traditionally been on elucidating the effects and applications of WBV to enhance musculoskeletal performance in athletes and individuals with musculoskeletal disorders, recent evidence highlights a noteworthy positive impact of WBV at the neurological level and brain functioning. This underscores its potential utility in addressing conditions related to brain diseases. Studies have been conducted to observe the effects of WBV on nervous system-related conditions like spinal cord injury, traumatic brain injury, stroke, anxiety, and major depressive disorder (20–23). Reviews on neurodegenerative disorders (discussed in Section 5) point toward a lack of optimized protocol. To start identifying optimized protocols for brain disorders, we believe that comprehending the molecular and cellular impacts of WBV is crucial.

**Figure 1:**
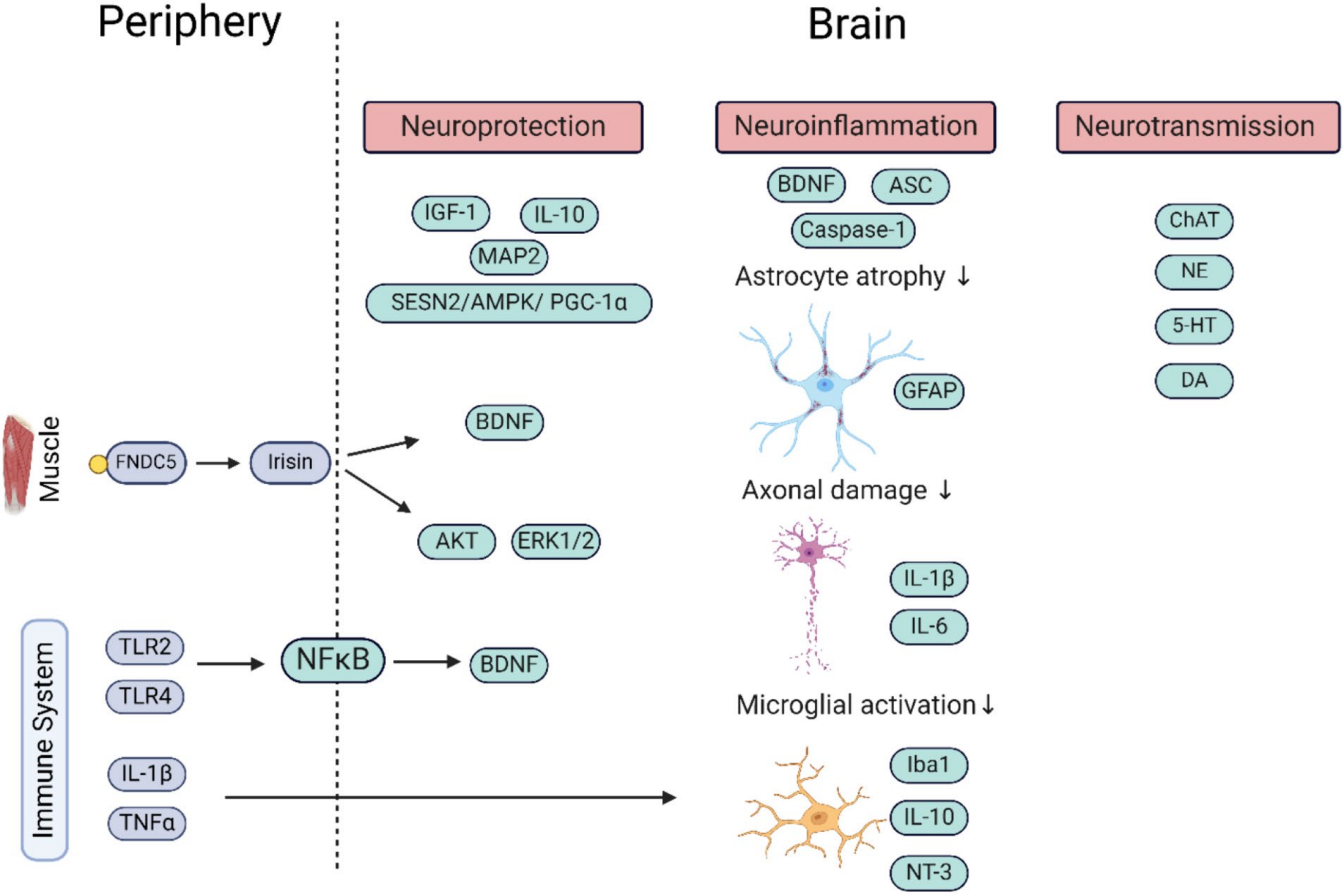
Schematic representation of the molecular pathways by which the periphery (factors in blue) influences the brain (factors in green) upon stimulation by WBV. More direct influences of WBV on the brain are due to only partly deciphered mechanisms. The factors secreted in the periphery (outside CNS) help regulate neuroprotection and neuroinflammation, and via stimulation of neurotransmitter system neurotransmission within neuronal networks. It shows the important role of the immune system in regulating brain functioning. The image was generated using Biorender. ASC, apoptosis-associated speck-like protein containing C-terminal caspase recruitment domain; AMPK, adenosine monophosphate-activated protein kinase; BDNF, brain derived neurotrophic factor; ChAT, choline acetyltransferase; DA, dopamine; ERK, extracellular signal-regulated kinase; FNDC5, fibronectin type III domain-containing protein 5; GFAP, glial fibrillary acidic protein; 5-HT, serotonin; IL, interleukin; IGF, insulin-like growth factor; Iba, ionized calcium-binding adapter molecule; MAP2, microtubule-associated protein 2; NFkB, nuclear factor kappa-light-chain-enhancer of activated B cells; NT-3, neurotrophin 3; NE, nor adrenaline; TLR, toll like receptor; TNF, tumor necrosis factor.

One of the key players in brain disorders is the immune system; it is known to be close-knit with the nervous system (24). Both these systems have a series of communication pathways to and from each other which play a role in maintaining the overall health of the body. On the one hand, the brain sends signals to the periphery via neurotransmitters, to get the immune system fired up, and on the other hand, the immune system sends signals back to modulate brain activity. This affects, among others, body temperature, sleep, and feeding behavior. In neurodegenerative disorders like Alzheimer’s disease (AD), Parkinson’s disease (PD), and amyotrophic lateral sclerosis, the immune system also plays a pivotal role, being both affected by and contributing to the regulation of disease progression. Cytokines such as in interleukins (IL)-IL-1B and IL6, tumor necrosis factor (TNFα), transforming growth factor (TGF)-TGFb1 and TGFα, along with immune-related factors like toll-like receptor (TLR)-TLR2 and TLR4, play key roles in this interplay. Proinflammatory and anti-inflammatory responses mediated by the immune system, along with oxidative stress, influence the delicate balance in neurodegenerative contexts (25, 26). Investigating how WBV impacts these immune responses holds promise for its therapeutic potential in immune-related conditions and its broader implications for neurodegenerative disorders. This review therefore aims to contribute to the effort to elucidate the intricate pathways influenced by WBV intervention, with a specific focus on molecular and cellular aspects linked to immune and brain functioning. We summarize what is currently known about the impact of WBV on the (brain) immune system and brain functioning, how peripheral effects influence the brain, and finally what is known about the impact on brain disorders. The literature selection criteria can be found in Supplementary Files (Methods). The molecular pathways affected by WBV are summarized in Figure 1, and Supplementary Tables 1, 2 have the molecular and cellular pathways in preclinical and clinical studies, respectively. We conclude with a brief discussion including suggesting aspects to consider while optimizing protocols based on underlying cellular and molecular mechanisms.

## 2 Immune system

The human immune system is not only responsible for combating infection by pathogens, exposure to environmental toxins and allergens, and cellular damage but also plays a pivotal role in maintaining homeostasis of the body (27). The immune system comprises a varied population of immune cells present throughout the body instead of being localized in an organ. Below, we will discuss the literature available reporting the effects of WBV on immune functioning. First, pre-clinical data will be discussed followed by studies that combine clinical and preclinical experiments, and finally, the clinical studies will be addressed.

### 2.1 Preclinical studies

Similar to exercise, WBV helps to decrease the inflammatory response as well as reverse symptoms of type II diabetes mellitus (T2DM) (28). Yu et al., have studied the effect of WBV on omental macrophages and the fecal microbiome that were isolated from mice that were subjected to 20 min of WBV per day for 4 weeks, with a frequency of 30 Hz and an amplitude of 3 mm (28). The analysis of macrophages from the abdominal adipose tissues revealed that WBV treatment resulted in a significant increase in M2 macrophage (counter-inflammatory function) count and it restored the IL-10 level in diabetic mice to the resting level in control mice. Since the differentiation of immune cells is often connected to the microbiota, the group of Yu and co-workers also performed an analysis of the microbiota of these mice in the presence and absence of WBV. The results showed that WBV remodels the alpha and beta diversity of the microbiome of the alimentary canal. Overall, these findings support the notion that WBV potentially alters the microbiota which prompts innate and mucosal immunity. This further produces anti-inflammatory responses and reverses the adverse consequences by down-regulating the hyper-inflammatory state.

Nowak et al., subjected 10 adult male Wistar rats to WBV on a vibrating platform, which generated vertical vibrations at the frequency of 50 Hz and amplitude of 2.5 mm for 5 weeks to determine the effects of WBV on hormonal and immunological parameters (29). Every session included four bouts lasting 30 s, separated by 1 min rest intervals. Following the training period, red and white blood cells, lymphocytes, monocytes, hemoglobin, granulocytes, and hematocrit, as well as IL-1b, IL-10, IL-6, and vascular endothelial growth factor levels were determined. The results showed a significant decrease in concentrations of IL-10 and a possible increase in IL-1b and IL6 in the blood serum, which are cytokines responsible for pro-inflammatory actions. However, a subsequent study done by the same group and the same WBV program showed no statistically significant changes in the complete blood cell counts or inflammatory cytokines (30). Interestingly, the only difference between the two studies was the period of WBV application. The later study showed the effect of WBV after three and 7 months of application, whereas the initial study presented the results for 5 weeks of WBV application. The disparity in the two studies shows that the total duration of WBV may be an important factor to take into consideration while studying the effects of WBV.

### 2.2 Combined preclinical and clinical studies

The study of Song et al., further strengthens the notion of WBV affecting the immune system through the change in the microbiota of the alimentary canal (31). The experimental setup of this study consisted of both mice and human volunteers who were subjected to 30 min of vibration every day for 30 days. Analysis of immunological markers revealed a significant increase of CD-4 and CD25 positive lymphocytes and CD4 and CD25 positive Treg cells in the spleen of the WBV-subjected mice. These immunological results suggest that WBV alters regulatory T-cell differentiation in mice. Furthermore, Song et al., also showed that WBV affects the intestinal microbiome of both humans and mice. WBV significantly reduced the alpha diversity in mice and increased the beta diversity of both mice and human fecal microbiota. Moreover, correlation analysis revealed significant changes in bacteria variation that are linked to regulatory T-cell differentiation in mice and physical characteristics in humans. This study thus also suggests that WBV has potential interventional effects on microbiota and immune-related diseases. The malleability of T cells in response to the gut microbiome can be used as a tool for editing immune response by utilizing microbiota-mediated pathways (32). Therefore, WBV-induced changes in microbiota, immune state, and inflammation of the body are research areas with clear potential and hence warrant further investigation.

### 2.3 Clinical studies

WBV has been tested as a treatment approach for osteoarthritis (OA) in several clinical studies. Systematic reviews have indicated the beneficial effects of WBV on pain, knee extensor muscle strength, and physical function in individuals with knee OA (33, 34). Even though OA has been considered to be a form of joint inflammation, there are now studies that indicate the implication of a systemic inflammatory response presented by T cells and the presence of inflammatory markers in peripheral blood, including inflammatory cytokines and antibodies (35, 36).

Since the immune system plays a causative and consequential role in OA, Tossige-Gomes et al., studied the effect of WBV, in addition to squat training, on the T-cell proliferative response of elderly subjects with knee OA (37). The patients were subjected to vertical synchronous vibration with a frequency ranging from 35 to 40 Hz, an amplitude of 4 mm, and an acceleration ranging from 2.78 to 3.26 g. The results showed that WBV decreases the proliferation of TCD4^+^ cells in patients with OA of the knee, suggesting that the addition of WBV to training might modulate T-cell-mediated immunity in this population, thereby minimizing the disease progression in elderly OA patients.

Chronic inflammation, a hallmark of aging, contributes to various age-related diseases. A recent study investigated how WBV impacts the inflammatory status in older subjects, focusing on TLRs 2 and 4 (38). The study included older individuals (average age 70 years) without significant health conditions. Participants underwent WBV sessions (30 Hz, 2 mm amplitude) for 12 weeks followed by blood sample collection before and after the intervention. TLR2 and TLR4 expression levels were measured, along with inflammatory marker IL-10, and physical performance (e.g., gait speed) was assessed. WBV led to a significant reduction in IL-10 levels, indicating an anti-inflammatory effect. TLR2 and TLR4 expression decreased after WBV, suggesting modulation of immune responses. These changes correlated with improved physical performance, emphasizing the holistic impact of WBV. Mechanistically, WBV likely influences TLR signaling pathways, and downregulation of TLR2 and TLR4 may contribute to the observed anti-inflammatory effects. The enhanced physical function may result from reduced inflammation. Lower TLR2 and TLR4 cell surface expression is frequently associated with the anti-inflammatory situation induced by a physically active lifestyle (39).

Also, the impact of WBV on circulating stem/progenitor cells (CPC) and cytokine levels has been studied. Healthy male participants engaged in three activities randomly: standing platform vibration, repetitive leg squat exercise, or a combination of both (40). The vibrations were subjected at 35 Hz with an amplitude of 4 mm. Blood samples taken before and after each activity revealed significant increases in CPC levels with exercise alone and vibration alone, particularly in younger subjects. Combined activity notably boosted angiogenic CPCs in younger participants. Vibration alone increased non-angiogenic CPCs in younger subjects, while exercise alone showed similar effects in older individuals. Additionally, WBV led to a significant increase in anti-inflammatory cytokine IL-10 and a decrease in inflammatory IL-6 levels. Notably TNFα and vascular endothelial growth factor levels increased with vibration alone, suggesting pro-angiogenic effects. The findings suggest WBV’s potential positive effects on vascular health and inflammation.

In contrast to a relatively large number of studies that showed the positive effect of WBV on the immune system, a few studies did not observe an effect of WBV on the immune response. For example, in one of these studies, the effect of WBV in combination with resistance exercise on salivary cortisol and salivary IgA was determined (18, 41). Nine adults were subjected to two bouts of resistance exercise with and without WBV of 30 Hz for approximately 18 min, spaced at a 7-day interval. No significant increase in salivary cortisol and IgA levels was found. However, further studies are required in this area with a higher sample size and focusing on the chronic effect of WBV rather than an acute effect. Another reason why WBV did not show a strong effect on the IgA secretion in this study, could be because of the use of a WBV platform that oscillates in the vertical direction only. It was reported that at frequencies of 25 and 30 Hz, the side-alternating platform produces twice the vertical acceleration in comparison to the vertical oscillation platform (42).

Taken together, WBV seems to play a role in modulating the immune response. By altering the gut microbiome in both humans and mice, WBV alters T-cell differentiation and induces a shift in M2 macrophages. Apart from regulation through the gut microbiome, WBV also alters proinflammatory markers. Next, we will discuss the impact of WBV on brain functioning.

## 3 Effects of WBV on the brain

Even though the initial focus of WBV treatment was to enhance muscular functioning, as the WBV applications advanced, its potential effects on the brain have become clear from various studies. The effects of WBV on brain functioning have been studied in healthy as well as patients and rodents including models for different neurological disorders or aspects of such disorders. First, the preclinical studies done with primarily mice and rats will be summarized, followed by clinical studies.

### 3.1 Brain functioning

#### 3.1.1 Preclinical

Multiple studies have shown that WBV also exerts effects on brain functioning. For example, exposure to WBV (30 Hz, 5 or 30 min per day for 5 weeks) has a positive effect on novel object recognition and motor performance in a motor test (balance beam performance) in CD1 mice (43). In another study, positron emission tomography in C57Bl6 mice revealed that glucose uptake was not changed as a consequence of the 5-week WBV intervention with 30 Hz for 10 min per day, 5 days a week (44), however, the arousal-induced home cage activity was reduced. These results suggest that WBV intervention improves motor performance and affects brain functioning in mice. Moreover, it can be suggested that WBV is a safe intervention to improve brain functioning, although the somewhat subtle effects suggest that the protocol is as yet suboptimal.

Aging can affect a person adversely, not only physically but also mentally. Physical exercise has been shown to have positive effects on mental well-being and the cognitive abilities of the elderly. Due to old age, people are often not able to exercise enough to get positive effects on functions such as neurological memory, anxiety, and motor performance (45). In such cases where physical exercise is not possible, WBV can be used as a form of passive exercise to achieve similar results as physical exercise.

In rodents, aging is associated with impaired memory functions (spatial and object) (46, 47), anxiety (48, 49), depression (48), and motor performance (50). Recent preclinical studies revealed that WBV for 5 weeks can affect the cognitive abilities of 18 and 30-month-old Wistar rats (20, 21). The rats were divided into two groups, vibration and pseudo-vibration groups. The vibration group was subjected to 5 weeks of mechanical vibrations at a frequency of 30 Hz and amplitude of 0.05–0.2 mm. The study with 18 months old rats used an intervention session of 10 min whereas the study with 30 months old rats used 5 min. Evidence from both these studies indicates that WBV can curtail anxiety, significantly improve the rearing behavior, and spatial memory of the rats, and also increase their motor performance. It is important to note that even brief daily sessions, lasting less than 10 min, could be enough to enhance memory functions and reduce anxiety-like behavior in advanced aging.

Another study, further discussed in Section 3.3.1, concluded that 20 min of stimulation results in a decrease in anxiety and an increase in spatial memory awareness. Five minutes of stimulation resulted in increased motor performance. Overall, it was shown that WBV can help improve both motor and cognitive functioning in 18-month-old Wistar rats. In contrast, from what has been found in old rats, anxiety-like behavior was reduced when 5 or 10 min sessions were used, but not when 20 min was used in 12-month-old female Wistar rats (51). Taken together, the data suggest that the sensitivity of the brain to WBV is age-dependent in rats.

#### 3.1.2 Clinical

In a recent clinical study exploring the effects of WBV, 133 young and healthy individuals (including 112 females and 21 males) with an average age of 20.5 ± 2.2 years underwent WBV treatment (52). The treatment involved exposure to mechanical vibrations at 30 Hz with an amplitude of approximately 0.5 mm for 2 min, repeated six times. The data revealed an enhancement in stroop color-word interference test scores, suggesting a positive short-term impact on executive functions, particularly on attention and inhibition, in young adults. Fuermaier et al., studied the effects of WBV on attention in 83 healthy individuals and 17 adults diagnosed with attention deficit hyperactivity disorder (ADHD) (53). Both healthy and adults with ADHD subjected to 30 Hz, 4 mm vibrations for 2 min showed small to medium effects on attention. Their results show that WBV may have potential as an alternative form of intervention to help with cognition in humans. An additional study investigated the impact of 3 min of WBV training at 30 Hz with an amplitude ranging from 0.44 to 0.6 mm on inhibitory function in healthy children (54). Their data showed a therapeutic effect associated with intelligence and age, though they did not specifically address ADHD. In healthy young adults, three bouts of two-minute side-alternating WBV (frequency 27 Hz) and three control conditions showed positive effects on cognition (55). The participants underwent two different sessions. In one session a sitting posture was used and in the other session a standing (semi-squat) posture. Their results showed that WBV significantly improved selective attention and inhibition in the sitting posture, but not in the standing posture. While significant, the effects were small.

A separate investigation examined the effects of WBV exercise on 12 healthy subjects using a frequency of 30 Hz and a 4-mm amplitude, administered for 2 min each over five sessions (56). Initially, the group had hypothesized that exposure to vibrations would decrease cognition. However, contrary to their hypothesis, they found that vertical vibrations increased motor processing speed. The outcomes indicated that WBV training, coupled with squats with a knee flexion at a 45° angle, did not significantly impact visual or verbal memory, reaction time, or impulse control, as assessed by the Immediate Post-concussion Assessment and Cognitive Test (ImPACT). However, there was a potential increase in motor processing speed following vertical vibration.

As mentioned earlier (Section 3.2.1), aging affects cognitive function and WBV can be a useful strategy to help keep an individual active. A study was conducted on elderly individuals where 17 people were randomly assigned to either an intervention group (*n* = 9) or a sham operation group (*n* = 8) (57). The intervention group underwent 4 weeks of WBV training for 1 min each, five times, 3 days per week. From weeks 5–8, a passive trampoline program of 5 min was introduced after the vibration sessions. The findings of this study showed that the eight-week program, combining stochastic resonance WBV and exergame-dance training, induced benefits in both physical and cognitive performance among older adults residing in care homes. Another study, which has been discussed above in the preclinical Section 3.1.1 also studied the effects of WBV on humans (44). Their protocol changed from mice to humans, in terms of the duration. Humans were treated with 30 Hz of vibration for 4 min per day for 4 days a week, for 5 weeks. They observed a positive effect in the older population in the stroop test, indicating improvements in selective attention and inhibition.

Since cognition is linked to the falls experienced in aging, Rosado et al., studied the effects of WBV in combination with a psychomotor intervention for 24 weeks (58). The vibration amplitude was always 3 mm and the frequency increased from 12.6 to 15 Hz. They tested the effects on reaction time, mobility, and dual-task performance in older adults at risk of falling. Their results showed improvements in reaction time, mobility, and dual-task performance in the group that had undergone psychomotor and WBV treatment. Notably, the influence of the interventions on reaction time, mobility, and dual-task performance was no longer apparent during the 12-week follow-up period without any additional intervention. Contrary to this, another study did not observe any improvements in fall risk, life satisfaction, and cognitive status in elderly women (59). The study consisted of 8 months of WBV intervention at 20 Hz with a 2-mm amplitude (30–35 min each, twice per week).

Two systematic reviews of 18 studies (including the ones mentioned above) revealed mixed results: some studies reported positive effects of WBV on cognition, while others found no significant impact (60, 61). Participants included both individuals with cognitive impairment and healthy subjects. The reviews concluded that WBV improved motor skills (56), reaction time (62), inhibitory function (54), processing speed, and executive functions in healthy individuals (52). The positive effects extended to patients with cognitive impairments as well. Studies showed improved attention, memory, and divergent thinking in ADHD patients (53, 63), and improved cognitive abilities in multiple sclerosis (64), dementia, and stroke patients (65, 66). The positive effects of dementia and stroke are both related to the activation of the cerebral cortex. Contrary to this, there were studies showing no change in WBV subjection in healthy and diseased subjects such as mild dementia (67). The majority of the studies indicated that WBV training enhances cognitive performance, with only a minority concluding otherwise. The disparities in results may be attributed to variations in the cognitive tests utilized, patients’ disease stages, and the protocols employed for vibration.

### 3.2 Neurotransmission

#### 3.2.1 Preclinical studies

Initial studies addressing the effect of WBV on the brain were mainly focused on determining the adverse effects of WBV on monoamines. Monoamine neurotransmitters like serotonin (5-HT), norepinephrine (NE), noradrenaline (NA), and dopamine (DA) play a pivotal role in the brain to further assist and regulate cognitive functions (68). They also play an important role in regulating systems other than the brain, like the cardiovascular system, respiratory system, and gastrointestinal system. Several studies have shown that an acute WBV exposure for 240 min stimulated the synthesis and release of several neurotransmitters and monoamines such as 5-HT (69, 70), NA (71, 72), corticosterone (70, 73), DA (69, 72), NE (72) and 5-hydroxy indole acetic acid (5-HIAA) (70). Although this thus suggests that monoamines are affected by WBV, it is important to note that all these studies were done with an extreme 4-h-long acute WBV session. The experimental design of these studies is inept as their vibration setup restricted the movement of the animal, the vibration protocols were very long and the results are unreproducible due to a lack of enough information like the age of the animals. Therefore, to get a better insight into the effects of WBV on these monoamines, and WBV’s effect in terms of therapeutics, it is essential to perform further studies that do not have such extreme durations, but rather use a few bouts of shorter periods and a reduced maximal acceleration with a properly designed setup.

Another study by Dmitriev et al., compared the effect of an acute WBV session to the effects of a 52–54 days long chronic WBV intervention with a frequency of 10 Hz, amplitude of 1 mm, and 15-min daily session duration (74). This vibration protocol enhanced the accumulation of 5-HT in different regions of the rat brain, especially in the hippocampus (acute) and the parietal complex (long-term). This study tried to explore the involvement of regional alterations in 5-HT metabolism and the responsiveness of serotoninergic structures in the development of somatosensory disorders linked to vibration exposure. These early studies together showed that WBV may help attenuate the levels of monoamines in the brain. To further test this hypothesis it would be interesting to study the effect of WBV in diseases where disbalance in these monoamines causes pathogenesis, for example in PD, AD, anxiety, seizure disorders, and mania. Notably, a recent study demonstrated that exposing rats to 80 Hz of WBV leads to the intracellular relocation of δ-opioid receptors from the cytosol to the membrane within rat cholinergic interneurons in the nucleus accumbens (75). This study primarily concentrated on the influence of WBV in mitigating dopamine-related mechanisms associated with morphine addiction. However, their findings also show the need for further investigation into the potential effects of WBV on dopaminergic neurons. Research could also hold considerable significance in the treatment of neurological conditions like PD.

As described previously, there is accumulating evidence that points toward a positive impact of WBV in rats and mice, on memory. However, the exact underlying mechanism by which WBV improves cognition and brain functioning remains largely unknown. To understand how WBV can affect the brain, it is important to consider its effect at a cellular and molecular level, for example by studying neurotransmitters and monoamines. It has been shown that the cholinergic forebrain plays a pivotal role in learning and memory performance (76). Five weeks of WBV stimulation in mice resulted in a significant increase in choline acyltransferase (ChAT), the rate-limiting enzyme for the production of acetylcholine. Since increased ChAT is strongly linked to increased cholinergic activity, this suggests that WBV treatment positively affects attention and memory through increased activity of the cholinergic system of the brain (77).

To recapitulate, studies indicate toward involvement of WBV in the attenuation of neurotransmitters like ChAT, NE, 5-HT, and DA (Figure 1). Although it is important to note that some of these studies have used extreme intervention protocols, therefore more research is needed in this field with better intervention protocols aimed at promoting health.

In a study led by Cariati and colleagues, they proposed that the impact of vibration training on cognitive processes might be age-dependent, and closely linked to synaptic plasticity (78). The study utilized mice of two different age groups, 4 months and 24 months. Synaptic plasticity, assessed through electrophysiological measures in the hippocampus, was investigated after exposing the mice to vibrations at 45 Hz for three series of 2 min and 30 s, with an equivalent recovery period in between, over 12 weeks. The outcomes showed a difference in response to the vibration in old mice compared to the young mice. Both age groups affected synaptic plasticity. Importantly, it is noteworthy to consider that the specific parameters of WBV, such as frequency and duration, might interact differently based on the age and possibly sex of the mice. This provides preclinical evidence and a good base point to continue these studies in aging humans who cannot perform physical exercise.

### 3.3 Neuroinflammation

While studying brain functioning, it is important to address neuroinflammation. Neuroinflammation is defined as an inflammatory response within the brain or spinal cord that is mediated by the production of cytokines, reactive oxygen species (ROS), chemokines, and secondary messengers (79). It essentially is a response of the central nervous system to disturbed homeostasis. Neuroinflammatory responses may be helpful or harmful, as mechanisms associated with neuroinflammation are involved in normal brain development, as well as in neuropathological processes. There are complex and interacting immune, biochemical, physiological, and psychological consequences of neuroinflammatory responses (79, 80). As neuroinflammation is increasingly recognized as being involved in nearly all brain disorders (81, 82), it is of critical importance to decipher the impact of WBV on neuroinflammation.

#### 3.3.1 Preclinical studies

Microglia, are the resident immune cells of the central nervous system that respond to insults and injuries in the CNS (83). Activation of microglia prompts the release of pro-inflammatory factors, contributing to neuroinflammation (84), in conditions like AD and PD. In SCI, microglia are a double-edged sword, aiding healing but potentially causing harm through the release of cytotoxic elements (85). In a recent study, rats with spinal cord injury (SCI) underwent WBV with an amplitude of 1.5 mm and frequencies of 15 Hz and 30 Hz (86). The therapy started on day 7, day 14, or day 28 post-injury, each with 10 rats. WBV sessions occurred 5 days a week, featuring five trials, with a one-minute rest between each. A control group had 10 rats with SCI but no additional therapy. Assessing functional recovery and immunohistochemical markers ionized calcium-binding adapter molecule 1 (Iba1) and neurotrophin-3 (NT-3) revealed that WBV initiated at day 14 led to the most significant overall recovery, with a moderate increase in Iba1 and the highest increase in NT-3. Moreover, in the case of traumatic brain injury (TBI), WBV has the potential to manage neuroinflammation by suppressing the activation of microglia (see also Section 3.5) (22, 87). Furthermore, Oroszi et al., (discussed in detail in 3.3.1) demonstrate that WBV results in a significant decrease in microglial activation in the Cornu Ammonis 1 (CA1) and dentate gyrus subregions in aged male rats (88). Taken together, these studies indicate that WBV attenuates microglial activation which probably helps in mitigating neuroinflammation.

Neuroinflammation is also one of the prevalent pathological occurrences in ischemic stroke (89). Studies have suggested microglial polarization modification as a prospective treatment strategy for ischemic stroke (90, 91). An animal study investigated the efficacy of WBV in reducing frailty and brain damage post-ischemic stroke in reproductively senescent female rats (92). The animals underwent 30 days of WBV (Frequency of 40 Hz; Amplitude not reported) treatment performed twice daily for 15 min each session, 5 days each week. The data revealed a significant depletion of inflammatory markers and infarct volume with significant increases in BDNF and tyrosine kinase receptor subtype B (Trk-B). Following post-ischemic WBV, protein levels of caspase-1, ASC, and IL-1β in the peri-infarct area decreased by 88% (*p* < 0.05), 57% (*p* < 0.05), and 148% (*p* < 0.05), respectively, compared to the control condition. Caspase-1 activates pro-inflammatory cytokines like IL-1β, contributing to neuroinflammation (93). ASC facilitates inflammasome assembly, further promoting inflammation in neurodegenerative diseases (94). They also observed an improvement in functional activity after inducing stroke via transient middle cerebral artery occlusion (tMCAO). This was done in middle-aged female rats that were treated with WBV as compared to the no-WBV group. These results suggest that WBV intervention may be a potential therapy to reduce post-ischemic frailty in old women after a stroke (92). Furthermore, the same research group also showed that in rat stroke models, WBV can protect against cognitive decline after undergoing a tMCAO (95) (more details in Section 4).

One example of a psychological disorder caused due to neuroinflammation is major depressive disorder (MDD) (96). Multiple studies in the context of MDD have indicated that exercise can deploy neuroprotection by enhancing synaptic plasticity, inhibiting apoptosis of neurons, ameliorating inflammation, and boosting the secretion of neurotrophic factors (97–99). Since lack of motivation to exercise and psychosomatic lethargy are intrinsic symptoms of MDD, establishing an exercise routine becomes challenging for the patients. In these cases, WBV can be used as a passive form of exercise that takes little time per day, especially since it also has been shown to beneficially alter depressive status in adolescents with depression (100). A preclinical study on the effects of 8 weeks of WBV intervention on neuronal loss, synaptic protein expression, and neurotrophic factors level in a rat model of chronic restraint stress-induced depression demonstrated that WBV significantly enhanced neuroprotection and recovery of degenerated neurons (101). The rat model was subjected to a vibration frequency of 30 Hz with an amplitude of 4.5 mm, 30 min per day, 6 days a week for 8 weeks, and behavioral and biochemical tests were performed. The authors hypothesized that the mechanism underlying the neuroprotection involves inhibiting the degeneration of neurons; inhibiting reactive microgliosis and astrocyte atrophy; protecting synapses, strengthening neural connections, as well as restoring impaired memory; reducing dendritic and axonal damage via microtubule-associated protein 2 (MAP2) and protecting damaged neurons from further damage; and enhancing the expression of trophic factors.

Oroszi et al., examined the dose-dependent effect of a 5-week-long chronic WBV intervention on anxiety-related behavior, memory, and motor functions, as well as a marker of (neuro)inflammation (88). Eighteen-month-old Wistar rats were stimulated by WBV for 5 or 20 min per day along with a control group with pseudo-WBV. After 5 weeks of WBV intervention, the anxiety-like behavior and motor performance were tested, which was then followed by brain analyses via immunohistological assays to determine hippocampal neuroinflammation. Both 5 and 20 min resulted in a significant decrease in microglial activation in the CA1 and dentate gyrus subregions.

In mice with myocardial infarction (MI), increased oxidative stress and inflammation can lead to harmful changes in the brain, causing nerve damage and neurodegenerative diseases (102). Sestrin 2 (SESN2) has been shown to alleviate oxidative stress and improve cognitive and cardiovascular health in aging and disease contexts (103). Overexpression of SESN2 activates the AMPK and PGC-1α pathway, enhancing cardiac function in aged mice and reducing cerebral ischemia/reperfusion injury in rats (104, 105). Feng et al., studied the effects of WBV on prefrontal lobe injury and dysfunction in mice with myocardial infarction (106). They focussed specifically on the SESN2/AMPK/PGC-1α signaling pathway. They revealed that WBV shows promising outcomes in alleviating prefrontal lobe injury and dysfunction. Specifically, the activation of the SESN2/AMPK/PGC-1α signaling pathway which mitigates oxidative stress and inflammation was identified as a key mechanism underlying this therapeutic effect.

In PD models, SESN2 upregulation triggers a reduction in neurotoxicity (107) and an autophagic response, preventing α-synuclein expression, apoptotic caspase-3 activation, and cytotoxicity in dopaminergic cells (108). In AD models, SESN2 induction counters amyloid beta (Aβ)-induced toxicity by promoting autophagy. These findings suggest that SESN2 may serve as a prognostic marker and therapeutic target in neurodegenerative diseases (109). Hence, exploring the impact of whole-body vibration (WBV) on SESN2 in the context of PD and AD could be intriguing.

In summary, WBV shows promise in alleviating neuroinflammation by reducing microglial activation in SCI and ischemic stroke models. It holds therapeutic potential for managing conditions such as MDD and anxiety. Additionally, it attenuates oxidative stress and neuroinflammation through the SESN2/AMPK/PGC-1α signaling pathway. The molecular pathways involved in neuroinflammation are summarized in Figure 1. However, despite promising preclinical evidence, the lack of clinical studies on neuroinflammation in human brain tissues limits our understanding of WBV’s efficacy in the clinics.

### 3.4 Neuroprotection

Neurotrophins or neurotrophic factors (NF) are proteins that are responsible for the survival, development, and function of neurons in both the central and peripheral nervous systems (110). Given their extensive influences on neurons, NFs are a good candidate for treating neurodegenerative and other neurological disorders (111). Some of these neurotrophins have been studied in the context of the effects of WBV, which will be discussed in this section.

#### 3.4.1 Preclinical studies

Insulin-like growth factor-1 (IGF-1) is a growth factor that is also classified as an NF. IGF-1 can cross the blood–brain barrier and stimulate protein synthesis in neurons, glia, oligodendrocytes, and Schwann cells, and favor neuronal survival while inhibiting apoptosis (112). Wu et al. found that WBV slows atherosclerosis progression in mice by regulating IGF1 (113). Mice underwent 12 weeks of WBV at 15 Hz for 30 min. It was shown that WBV significantly reduced atherosclerotic plaque area and exhibited decreased serum IGF-1 and lower expressions of IL-6, IGF-1R, and p-IGF-1R protein in the aorta. Interestingly, serum IGF-1 peaked 30 min post-WBV for durations of 10, 30, 60, and 120 min. This suggests that appropriately timed WBV may impede atherosclerosis progression, associated with acute serum IGF-1 elevation and sustained lower aortic IGF-1 and IL-6 levels. Furthermore, Li et al., showed that in mice WBV also shows promise in promoting hypertrophy through mechanisms involving signaling pathways related to muscle growth such as IGF-1/IGF-1R–PI3K/Akt signaling (114). Moreover, the study by Peng et al., discussed above (Section 3.3.1) showed significantly increased levels of BDNF and IGF-1 in the hippocampus upon WBV training (101). This was not only associated with a protective effect on nerves and synapses but also resulted in an improvement in depression-like behavior in rats. Together, it can be concluded that WBV might be affecting the brain functioning via IGF. However, it has to be noted that only one of these studies analyzed whether the WBV-induced increase of IGF affects neural cells and/or brain functioning. Further investigating the neuronal effects of WBV-induced IGF might help to understand the role of WBV in neuroprotection, plasticity, and regeneration.

A study compared the effects of active exercise (treadmill running) and passive exercise (WBV) post-surgery (115). Rats underwent abdominal surgery, followed by active or passive exercise for 14 days. WBV protocol consisted of 30 Hz, 0.05–0.2 mm. The duration of WBV was 10 min on the first-day post-surgery and twice a day for 10 min from the second day onwards. The two vibration treatments were 6 h apart. Both active and passive exercise improved cognitive flexibility and memory was not affected by either treatment. Muscle strength increased upon active exercise but was unchanged by WBV. After the sacrifice, neuroinflammation was studied by observing microglial activation and neurogenesis by doublecortin (DCX) staining. Results showed no impact on surgery-induced inflammation but the treatment induced neurogenesis in the hippocampal region after both kinds of exercises. It is noteworthy that the expression of BDNF and IGF induces neurogenesis via DCX (116, 117). The results from this study may suggest that exploring these three factors may give better insight into the neurogenesis effects of WBV by studying.

The major NF brain-derived neurotrophic factor (BDNF) plays various important roles in the functioning of the brain. It is essential for brain development due to its involvement in differentiation, migration, and neuronal survival, and it exerts a role in dendritic development and in regulating synapse genesis and plasticity (118–121). Consequently, BDNF is fundamental for hippocampal functioning and learning (120–123).

In post-ischemic mice, following 4 weeks of vibration therapy, increased levels of BDNF were observed (92). MPTP (1-methyl-4-phenyl-1,2,3,6-tetrahydropyridine) lesion mice, a model to study PD that were subjected to WBV, showed increased levels of BDNF and consequently a protective effect of dopaminergic neurons (124). In addition, Peng et al., have also shown that WBV might be protecting the neurons via alleviating BDNF levels in the hippocampus of MDD rodents (101). Similarly, young mice showed increased BDNF expression in their cerebellum and hippocampus after 3 months of vibration exposure, 3 days a week (125). Furthermore, they showed that WBV also can increase FNDC5 expression, which in turn further increases BDNF and thereby results in better musculoskeletal functions via myostatin and collagen I. Interestingly, FNDC5 expression upon exercise is directly linked to the functioning of irisin, which will be discussed in detail in Section 4.

#### 3.4.2 Clinical studies

There is inconsistency amongst the limited clinical studies that have been done on testing the effect of WBV on IGF. There are clinical studies that have seen no significant effects on serum IGF levels in human subjects after WBV subjection (126–131), while a study in elderly individuals shows that WBV affects the levels of IGF-1 (132). Participants experienced two interventions with a minimum 2-week gap: vibration and control (no vibration). In both interventions, individuals stood on a vibration plate with a slight knee flexion, undergoing five 1-min sessions separated by 1-min rest periods. For the vibration intervention, the plate vibrated at a frequency of 30 Hz with a 4-mm amplitude. This group observed an acute increase in the circulating levels of IGF-1 and cortisol in elderly individuals to a greater extent than an exercise protocol conducted without vibration.

Concerning BDNF, it has been shown that a single session of vibration with a frequency of 35–40 Hz, amplitude of 4 mm, and acceleration gravity ranging from 2.78 to 3.26 g exerts no effects of WBV on BDNF levels in women with fibromyalgia (133). In contrast, the same researchers reported in 2021 that WBV treatment for 6 weeks can increase the expression of BDNF in women with fibromyalgia (134). Additionally, it leads to improved lower limb muscle strength, aerobic capacity, clinical symptoms, and QOL after being subjected to vibration with the same vibration settings as the previous study. The only difference between the two studies was the duration of WBV intervention. The later study showed positive effects on BDNF levels after 6 weeks of WBV, whereas the 2018 study was based on a single exposure to WBV. This indicates that a longer – chronic intervention might be beneficial over a single-acute session of WBV.

Studies on elderly women with knee osteoarthritis suggest that the observed improved lower limb muscle performance after WBV might be mediated by an increase in BDNF levels in the serum (135). Their experimental setup consisted of 12 weeks of WBV intervention with a frequency of 35–40 Hz, amplitude of 4 mm, and acceleration that ranged from 2.78 to 3.26 g. In contrast, other studies have reported no apparent effect of WBV on BDNF levels in depression, spinal cord injury, and even in young, healthy women (131, 136, 137). Wunram et al., used a training program for individuals with depression that encompassed six different types of standardized exercises, with each exercise lasting 2 min at a frequency of 20 Hz and an amplitude of 2 cm, conducted over 6 weeks (131). They failed to see an effect of WBV on BDNF levels with this WBV protocol. However, as acknowledged by the authors, this study has some limitations, including a relatively small sample size, the absence of randomized controls, and the omission of psychosocial factors from consideration.

In a study that focussed on patients with spinal cord injuries, the vibration platform operated at 35 Hz with a 2 mm vertical displacement. During the training, participants engaged in fifteen 1-min bouts of vibration interspersed with 1-min rest intervals (136). As all participants had chronic spinal cord injuries, it remains uncertain whether a single bout of WBV would induce significant acute increases in BDNF in individuals with chronic injuries. Additionally, a single exercise session might not have provided an adequate stimulus to elicit an acute neurotrophic response. Furthermore, even in the case of young and healthy women who experienced vibrations with amplitudes of 2 and 4 mm and frequencies ranging from 20 to 60 Hz (137). These women participated in individually supervised sessions three times a week for 3 months. No significant effects were observed as an effect of WBV.

In conclusion, WBV can contribute to neuroprotection through neurotrophins like IGF-1, BDNF, and IL-10 (Figure 1). WBV increases FNDC5, a precursor protein of irisin which further increases BDNF (discussed in more detail in Section 4). Therefore, by investigating how WBV impacts neurotrophin expression and function, we may uncover novel strategies for enhancing brain health and promoting neuroprotection, which could pave the way for personalized approaches to maintaining cognitive well-being.

Previously, we have structured our sections into preclinical and clinical delineations. However, henceforth, we will refrain from such categorization for the forthcoming sections, as they lack relevance within these frameworks.

## 4 Irisin, a possible link between the effect of WBV in the periphery to the brain?

Irisin is a relatively newly discovered exercise-induced myokine, which is involved in the regulation of several bodily processes such as glucose homeostasis, reduction of systemic inflammation, and modulation of energy metabolism through the browning of white adipose tissue (122). It is secreted by muscles in response to exercise (138) or WBV in humans (139, 140).

To understand how WBV may affect the brain through irisin production, it is important to know how irisin is produced. It is known that exercise induces an increase in calcium signaling which activates the transcriptional co-activator PPAR-γ co-activator-1 α (PGC1-α), which subsequently augments the expression of type 1 membrane protein, FNDC5 which is then cleaved to irisin (141). The impact of irisin on cognition is to a large extent elicited by the induction of BDNF expression. Post-exercise FNDC5 mRNA is upregulated in the hippocampus, which coincides with BDNF expression (122).

Since BDNF is essential for various brain functions and is implicated in exercise-induced cognitive benefits (142), parallel upregulation of FNDC5 and BDNF mRNAs in hippocampal neurons following exercise might suggest that irisin has a role in neuronal survival, activity, and cognitive functions.

A study in mice demonstrated that by regulation of the Akt and ERK1/2 pathway, irisin protects neuronal cells (PC12) from ischemic injury, suggesting that irisin may be a factor linking metabolism and cardio-cerebrovascular diseases (143). A recent study carried out on stroke rat models, showed the potential use of WBV in protecting against cognitive decline after going through a transient middle cerebral artery occlusion surgery (tMCAO) (95). Treating rats with the frequency of 40 Hz (amplitude not reported) for 15 min twice a day for 1 month after tMCAO significantly reduced the cognitive deficit in rats. It was speculated that this protective nature of WBV could be because of increased serum levels of irisin and decreased proinflammatory cytokines.

A recent review discussed the role of exercise-induced irisin in improving mental health in type 2 diabetes mellitus (144). Briefly, it has been shown that patients with diabetes mellitus experience a decline in cognitive function and memory loss (145–147). Wang et al., assessed whether irisin has a positive effect on memory and cognitive performance in a diabetic mouse model (148). They induced streptozotocin to establish a diabetic mouse model in 8-week-old male C57Bl/6 mice and assessed cortical and spatial memory through novel object recognition tasks and the Y-maze spontaneous alteration task (to determine short-term memory of 8 min). Upregulation in the levels of glial fibrillary acid protein (GFAP), a biomarker for astrocytes, reduction in synaptic protein expression, and an increase in the levels of IL-1β and IL-6 was observed. They also observed inhibition in the activation of proteins responsive to stress stimuli, like P38, STAT3 (Signal transducer and activator of transcription 3), and NFkB, in the diabetic mice. The reduction of cognitive function and memory which was observed in the diabetic mice could be avoided by irisin treatment, suggesting that an increase in irisin levels can improve and avoid the decline in cognitive function in diabetic mice.

Also, in clinical trials and studies, the effect of acute and chronic WBV training on circulating irisin levels has been evaluated (140). Young, healthy, untrained females were subjected to a 6-week program of WBV training with two sessions per week. The training regime consisted of seven different isometric exercises (different types of squats, elbow dips, and triceps exercises) in combination with a vibration frequency of 16, 19, and 21 Hz (increased during training) and an amplitude of 2.5 mm during the first 4 weeks and 5 mm during the last 2 weeks. The duration of each training session was progressively increased every 2 weeks from 11 to 18.5 min. Blood was drawn before and immediately after an acute bout of exercise at baseline and after 6 weeks of training. The resting irisin levels were not different at baseline and after 6 weeks of training, whereas an acute bout of vibration exercise significantly elevated circulating irisin levels by 9.5 and 18.1%, respectively at both 0 and 6 weeks of training. These findings indicate that acute bouts of WBV exercise can increase circulating irisin levels.

Moreover, a separate clinical study examined the influence of WBV on oxidative stress markers and irisin blood levels in women with fibromyalgia (149). WBV was subjected three times per week for 6 weeks, involving dynamic squats on a synchronized vibrating platform. The mechanical stimulation parameters included a vibration frequency of 35–40 Hz and an amplitude of 4 mm. The findings revealed that WBVT resulted in a reduction of visceral adipose tissue, an elevation in blood irisin levels, and a decrease in blood levels of the oxidative stress marker thiobarbituric acid.

Apart from its function in improving cognitive function and memory loss in diabetes, irisin has also been shown to have a potential role in treating AD (150). This study implied that the neuroprotection of irisin was mediated by blocking the release of Il-1*β* and IL-6 from cultures of astrocytes instead of its direct action on neurons. Their results also suggested the importance of the NF*κ*B signaling pathway in the regulation of irisin on astrocytes exposed to A*β*.

Although there are a lot of studies indicating the positive effect of the exercise-induced increase in irisin activity on cognitive functions and neuroprotection, however, there are very few studies done to specifically study the link between WBV and irisin. Further studying the effect of WBV on irisin activities might give a better insight into standardizing WBV protocols to exploit the beneficial effects of irisin upregulation for people who cannot indulge in physical exercise.

## 5 Brain disorders

Recently, a systematic review revealed the potentially beneficial effects of WBV for brain disorders using animal models but also stressed that WBV as a form of therapy needs further development (151). Therefore, in this segment, studies conducted on animal models or patients with various brain-related disorders will be discussed only briefly.

A common problem with aging is increased frailty which increases the risk of falls that subsequently can cause TBI (152). TBI can also be caused due to reasons that are independent of age factors like assault, motor vehicle accidents, incidents related to sports activities, or any other accidents that cause an injury to the brain. A preclinical study in mice indicates that WBV treatment can be an ideal treatment for patients suffering from TBI (22). In the case of TBI, 30 Hz of WBV for 20 days can reduce neuronal damage and improve cognitive and functional outcomes after TBI. This study shows that WBV: (1) alleviates cortical edema, (2) suppresses microglial activation, (3) inhibits GFAP expression that relates to astrocyte activation, (4) prevents the increase of IL-1β, TNFα, and IL-6 and promotes the increase of IL-10 which is an anti-inflammatory cytokine, (5) facilitates neuronal apoptosis, (6) improves exploratory behavior and general activity, (7) decrease learning and memory deficit after TBI, and (8) augment learning and memory deficit caused by TBI. Another preclinical study assessed the effects of WBV on induced brain injury (subarachnoid hemorrhage) in mice (87). Mice underwent WBV twice daily for 20 days at a frequency of 30 Hz. Their findings indicate that WBV decreases apoptosis, and moderates the heightened expression of GFAP (astrocyte marker) and Iba-1 (microglia marker). Additionally, WBV alleviated the loss of neurons in the hippocampus.

In contrast to the findings in preclinical studies, a clinical study done on individuals with stroke or traumatic brain injury showed no difference between the control group and the WBV group right after training for the first time or even after 2 weeks of rehabilitation therapy (153). The reason could be the very low frequency of vibration therapy. They used a frequency of 5 Hz for 1 min, which seems to be too little for the vibration therapy to have any effects. Therefore, this study should be performed again with a higher frequency (around 30 Hz) for a longer period than 1 min (ranging from 10 to 30 min).

Strokes are another form of neurological condition that can be caused due to an acute vascular injury. The potential application of WBV on stroke has been discussed in detail in Section 3.2. Some systematic reviews have indicated that based on the available data, no significant conclusions about the positive effects of WBV on stroke can be made yet (23, 154). However, there is clear evidence that WBV can be beneficial in the rehabilitation programs of patients and animal models after stroke by modulating spastic hypertonia, BDNF signaling, proinflammatory cytokines, and irisin production (92, 95, 124, 141).

Moreover, recent reviews have also discussed the effects of WBV on neurodegenerative disorders like AD (155, 156) and PD (157, 158). A review consolidating available evidence on the effects of WBV on AD demonstrates the effectiveness of WBV in enhancing neuromuscular function, functional mobility, and quality of life metrics in individuals with AD (156). It has also been shown that WBV as an intervention is feasible in fragile AD patients (155). Moreover, WBV ameliorates disrupted brain networks and various cognitive functions, including orientation, memory, and linguistic skills in AD patients. However, according to this review, the evidence for the effects of WBV on AD pathology seems inconclusive. Similarly, in a review previously conducted by our group, we found that the impact of WBV on the cognitive and brain function of individuals with PD is presently inconclusive (157). Nevertheless, to improve motor function of PD patients promising outcomes are evident in WBV protocols lasting at least 3 weeks, with a frequency of at least three sessions per week and vibration frequencies equal to or exceeding 20 Hz (157). These protocols show potential benefits, especially in enhancing motor function. Another recent review for PD has demonstrated that there are potential advantages of using WBV over conventional therapy but these need to be studied more (158). Overall, in the context of neurodegenerative disorders, most reviews are at a consensus regarding the need for an optimized protocol. AD and PD are the most prevalent neurodegenerative diseases. In 2022, we published a systematic review discussing the potential of WBV in PD (157). Through a thorough meta-analysis, we concluded that WBV has a significant but minor effect on motor and non-motor outcomes in PD. The promising results of studies demonstrating the ability of WBV to reduce neuroinflammation and oxidative stress in PD patients underscore the need for further research into the potential therapeutic effects of WBV. Coming to AD, we also published pre-clinical data which indicates that WBV may have beneficial effects on the early progression of brain pathology, particularly in restoring the morphology of GFAP-positive astrocytes to levels resembling those observed in non-pathological or “healthy” conditions (159). Another recent study in mice has demonstrated that daily treatment with 40 Hz WBV stimulation over multiple weeks led to a decrease in phosphorylated tau accumulation and neuronal loss (160).

## 6 Discussion

WBV has gained increasing attention in recent years due to its potential benefits in a wide range of health-related conditions. WBV possesses effects on the nervous system, ranging from improving balance, motor coordination, and neuromuscular function to cognitive functions, making it beneficial for those with neurological conditions. Here, we have summarized the effects of WBV on the cellular and molecular pathways involved in the immune system and brain functioning. As the effectiveness of WBV as a therapeutic intervention for the brain is still a matter of debate, these insights could aid in identifying more optimized WBV protocols.

WBV appears to influence the immune system by modulating T cell differentiation and inducing a shift in M2 macrophages through alterations in the gut microbiome. Additionally, it impacts proinflammatory markers, collectively suggesting a regulatory role in the immune response. The current review has also pointed toward the possible beneficial effects of WBV in various neurological disorders through cellular pathways such as neuroinflammation, neuroprotection, and neurotransmission. At a molecular level, WBV was involved in the regulation of inflammatory biomarkers IL-1B, IL-6, IL-10, CRP, TNFα, neurotransmitters such as Acetylcholine, NE, dopamine and 5-HT, neurotrophic factors like TNFR-1, TNFR-2, IGF-1, and BDNF (Figure 1). WBV seems to be regulating neuroinflammation and neuroprotection via SESN2/AMPk/PGC1a pathways. Moreover, BDNF regulates neuroinflammation, synaptic plasticity, and neuroprotection. Secretion of BDNF was found to be linked to the secretion of irisin, a myokine that gets secreted in the periphery. The available data warrant the importance of studying this molecule further as it shows a potential to be used as a key player for WBV studies along with BDNF. Moreover, to fully understand the effect of WBV on brain functioning at a molecular level, it is important also to study the link between additional factors that get produced by the muscles and which regulate the expression of proteins involved in brain functions such as attention, motor performance, memory, and learning.

Despite the potential benefits of WBV, there are several limitations to consider. One of the main challenges is the lack of standardized protocols for WBV, with considerable variations in the vibration frequency, amplitude, and duration used in different studies. Our review shows that most studies use frequencies in the range of 15–30 Hz, while only a few studies have used 40 Hz and 90 Hz vibration frequencies. It is important to note that the applied frequency is usually transmitted 1:1 to the brain (161). Brain entrainment via WBV is one way to achieve gamma stimulation (around 40 Hz), an increasingly used technique to promote brain health and to be used in the management of neurodegenerative disorders (162). Hence, a frequency of 40 Hz is to be advised from this point of view. Most studies vary in time of subjection. It was observed that using a protocol where the vibrations are subjected for a given time, followed by a rest period, and then subjecting the vibration again for the same amount of time may have the most beneficial effect. However, this needs to be confirmed more and for different conditions. Moreover, although poorly studied in SCI patients, a study shows that there is a window of opportunity, or critical period, during which WBV should be started after SCI for its beneficial effects (163). This aspect should be taken into consideration to extrapolate more beneficial effects of WBV; WBV effects concerning brain disorders may highly depend on the state of the disease. Populations studied so far have been relatively small and heterogeneous, with differences in age, gender, and health status. These limitations make it challenging to draw definitive conclusions about the specific molecular mechanisms underlying the effects of WBV and the potential applications of this intervention for neurological and neurodegenerative disorders. There is still a limited understanding of the specific mechanisms by which WBV may affect brain functioning. This review is a step forward in summarizing these mechanisms, and to stimulate research into that direction. Further research is needed to elucidate the mechanisms mentioned in this paper in more detail and to develop optimized and standardized protocols that can be used across different populations and conditions. Highlighting the critical need for methodological standardization in WBV research, it is important to note the lack of standardized processes in reporting available data within reviews as well. The current way by which literature reviews address the various parameters of the used WBV protocols could be improved if reporting guidelines for these parameters are followed. Based on the reporting guidelines proposed by van Heuvelen et al. (1), we have compiled a set of guidelines recommended for use in writing reviews (see Table 1). This consolidation aims to standardize the reporting of studies and ensure a more systematic and comprehensive approach to compare published WBV studies, thereby enhancing the chance of identifying the essential components determining the outcome of the many different WBV protocols and devices used in the field.

**Table 1:** WBV-specific aspects to consider while reporting about WBV studies in a review.

Aspect	Details to consider to extract from the selected papers and to discuss
Device	Manufacturer and type, specifications in case of self-built device
Type of vibration	Spatial and temporal characteristics (e.g., vertical or side-alternating)
Vibration parameters	Settings of the vibration parameters (frequency and magnitude) considering used definitions. Whether frequency and magnitude were constant or modulated
Verification of vibration parameters	Whether vibration parameters were manufacturer settings or verified, and, in case of verified, the methods used
Administration	Posture, position of feet and hands and footwear during vibration. Additional tools used. General exercise parameters (e.g., number of sessions, number of bouts, bout duration, rest intervals)
Control	If applicable: control condition or control intervention (e.g., sham WBV, no intervention/rest)
Population characteristics	Clinical studies: general characteristics and previous experience with WBV. Preclinical studies: used strains (e.g., the genetic background) and specifics of types of cells

For a detailed overview, see van Heuvelen et al. (1).

Taken together, to further unravel the intricate cellular and molecular mechanisms underlying the effects of WBV, a comprehensive approach combining multiple experimental strategies can be employed. Initial investigations can begin with cell culture studies, which offer a cost-effective and feasible method for subject-specific cell types to vibrations and analyze molecular changes. Utilizing both simple model organisms and disease-specific cell lines allows for the examination of WBV effects at a cellular level. Exploring WBV through diverse model organisms and examining its interconnected effects on various organ systems (e.g., muscles and brain) could yield a more comprehensive understanding of its impact on human health. Instead of narrowly focusing on individual aspects, a holistic system biological approach encompassing different species and organ systems could provide valuable insights. So far, the effects of WBV have not been studied with multiple systems and a multi-modal system approach. Studying WBV across various species can help elucidate its broader physiological and genetic implications. By considering its multifaceted effects, we may uncover new applications and therapeutic potentials that extend beyond isolated areas of research. Such interdisciplinary investigations could contribute to a more effective utilization of WBV for improving human well-being.

Moving toward a more holistic perspective, animal studies become instrumental in collecting tissue samples from various organs. These samples serve as valuable resources to conduct gene expression profiling using microarray analysis or RNA sequencing, proteomics employing mass spectrometry and Bio-ID technologies, and metabolomics analysis. These techniques provide insights by comparing control and WBV-treated samples, revealing differentially expressed genes, altered proteins, and affected metabolic pathways. To further validate the findings, molecular alterations can be examined using immunohistochemistry with microscopy tools and western blotting. By integrating these experimental approaches, a deeper understanding of the molecular mechanisms underlying WBV effects can be achieved. Once the specific proteins and genes involved in WBV effects are identified, these can potentially be used as basic read-outs in WBV research that may help establish a disease-specific or an individual-specific vibration protocol.

While conducting this review, several limitations were encountered that warrant discussion. Firstly, a limited amount of data is available on the molecular effects of WBV related to brain functions, which constrains a comprehensive understanding of its cellular impacts. Additionally, comparing conditions across different studies proved challenging because many papers lacked clear and detailed descriptions of the parameters used, despite existing older and newer reporting guidelines (1, 164). This inconsistency in reporting makes drawing reliable conclusions difficult. Furthermore, some studies present conflicting data, adding another layer of complexity. Although investigating the effects of WBV on other organ systems and their links to the brain would have been informative and could have enhanced the complexity of this review, the previously mentioned limitations such as unclear parameters, and a scarcity of studies exploring the crosstalk between different organ systems and the brain made it difficult to address this issue. At the moment, excluding the other systems does not diminish the value of our review, but in the future, when more data is available, it would be interesting to establish whether a WBV-sensitive link exists between the brain and the other organ systems.

The current review has many notable strengths. The review aims to raise awareness about the importance of carefully selecting protocols based on underlying mechanisms, which could help advance the field in a meaningful direction. So far no reviews have been published that focus on the molecular mechanisms involved in the effects of WBV on the brain and immune system together. Since the brain is very closely linked to the immune system, highlighting the importance of cross-talk between the two systems makes this study crucial and novel. Furthermore, the review includes a comprehensive range of studies (from cellular, preclinical, to clinical) that distinguishes this review from many others that have not covered such a diverse array of research. Furthermore, based on the available literature, this review provides several recommendations for conducting further research and improving data reporting, also for future reviews.

In conclusion, this review has consolidated the available data on the cellular and molecular effects of WBV on brain and immune functioning. The review emphasizes the importance of studying molecular mechanisms to design more optimal protocols.

## Author contributions

GA: Writing – review & editing, Writing – original draft, Methodology, Investigation, Conceptualization. YA: Writing – review & editing. MH: Writing – review & editing. AK: Writing – review & editing, Writing – original draft, Conceptualization. TO: Writing – review & editing. EZ: Writing – review & editing, Writing – original draft, Conceptualization.

## References

[1] Van Heuvelen MJG Rittweger J Judex S Sañudo B Seixas A Fuermaier ABM. Reporting guidelines for whole-body vibration studies in humans, animals and cell cultures: a consensus statement from an International Group of Experts. Biology. (2021) 10:965. doi: 10.3390/BIOLOGY10100965 34681065 PMC8533415

[2] Oroszi T van Heuvelen MJG Nyakas C van der Zee EA. Vibration detection: its function and recent advances in medical applications. F1000Res. (2020) 9:619. doi: 10.12688/f1000research.22649.1 PMC7308885 32595943

[3] Rittweger J. Manual of vibration exercise and vibration therapy. NYC, USA: Springer. (2020). 389 p.

[4] Hill PSM. Vibration and animal communication: a review. Integr Comp Biol. (2001) 41:1135–42. doi: 10.1093/ICB/41.5.1135

[5] van der Zee EA. The biology of vibration In: Rittweger J, editor. Manual of vibration exercise and vibration therapy. Cham: Springer (2020). 23–38.

[6] Horowitz S. The universal sense: how hearing shapes the mind. USA: Bloomsbury (2012).

[7] Guignard JC. Human sensitivity to vibration. J Sound Vib. (1971) 15:11–6. doi: 10.1016/0022-460X(71)90354-3

[8] Sá-Caputo D Taiar R Martins-Anjos E Seixas A Sartório A Sanudo B. Does the mechano-biomodulation vibration lead to biological responses on human beings? Ser Biomech. (2023) 37:3–17. doi: 10.7546/SB.01.02.2023

[9] Charles LE Ma CC Burchfiel CM Dong RG. Vibration and ergonomic exposures associated with musculoskeletal disorders of the shoulder and neck. Saf Health Work. (2018) 9:125. doi: 10.1016/J.SHAW.2017.10.003 29928524 PMC6005913

[10] Krajnak K Riley DA Wu J McDowell T Welcome DE Xu XS. Frequency-dependent effects of vibration on physiological systems: experiments with animals and other human surrogates. Ind Health. (2012) 50:343. doi: 10.2486/INDHEALTH.MS1378 23060248 PMC4694567

[11] Sitjà-Rabert M Rigau D Fort Vanmeerghaeghe A Romero-Rodríguez D Bonastre Subirana M Bonfill X. Efficacy of whole body vibration exercise in older people: a systematic review. Disabil Rehabil. (2012) 34:883–93. doi: 10.3109/09638288.2011.626486 22225483

[12] Kennis E Verschueren SM Bogaerts A Van Roie E Boonen S Delecluse C. Long-term impact of strength training on muscle strength characteristics in older adults. Arch Phys Med Rehabil. (2013) 94:2054–60. doi: 10.1016/J.APMR.2013.06.018 23831385

[13] Rogan S de Bruin ED Radlinger L Joehr C Wyss C Stuck NJ. Effects of whole-body vibration on proxies of muscle strength in old adults: a systematic review and meta-analysis on the role of physical capacity level. Eur Rev Aging Phys Act. (2015) 12:12. doi: 10.1186/S11556-015-0158-3 26865876 PMC4748331

[14] Marín PJ Herrero AJ Milton JG Hazell TJ García-López D. Whole-body vibration applied during upper body exercise improves performance. J Strength Cond Res. (2013) 27:1807–12. doi: 10.1519/JSC.0B013E3182772F00 23085972

[15] Jo NG Kang SR Ko MH Yoon JY Kim HS Han KS. Effectiveness of whole-body vibration training to improve muscle strength and physical performance in older adults: prospective, single-blinded, randomized controlled trial. Healthcare. (2021) 9:652. doi: 10.3390/HEALTHCARE9060652 34072657 PMC8226869

[16] Teles MC Fonseca IAT Martins JB De Carvalho MM Xavier M Costa SJ. Comparison between whole-body vibration, light-emitting diode, and cycling warm-up on high-intensity physical performance during sprint bicycle exercise. J Strength Cond Res. (2015) 29:1542–50. doi: 10.1519/JSC.0000000000000780 25764492

[17] Ardiç FN Alkan H Tümkaya F Ardiç F. Effectiveness of whole-body vibration or biofeedback postural training as an add-on to vestibular exercises rehabilitation therapy in chronic unilateral vestibular weakness: a randomized controlled study. J Vestib Res. (2021) 31:181–90. doi: 10.3233/VES-190753 33459675

[18] Wadsworth D Lark S. Effects of whole-body vibration training on the physical function of the frail elderly: an open, randomized controlled trial. Arch Phys Med Rehabil. (2020) 101:1111–9. doi: 10.1016/J.APMR.2020.02.009 32145279

[19] Chawla G Azharuddin M Ahmad I Hussain ME. Effect of whole-body vibration on depression, anxiety, stress, and quality of life in college students: a randomized controlled trial. Oman Med J. (2022) 37:e408. doi: 10.5001/OMJ.2022.72 36052109 PMC9396709

[20] Oroszi T Geerts E de Boer SF Schoemaker RG van der Zee EA Nyakas C. Whole body vibration improves spatial memory, anxiety-like behavior, and motor performance in aged male and female rats. Front Aging Neurosci. (2022) 13:962. doi: 10.3389/FNAGI.2021.801828/BIBTEX PMC8815031 35126091

[21] Oroszi T Felszeghy K Luiten PGM Schoemaker RG van der Zee EA Nyakas C. Whole body vibration ameliorates anxiety-like behavior and memory functions in 30 months old senescent male rats. Heliyon. (2024) 10:e26608. doi: 10.1016/J.HELIYON.2024.E26608 38404823 PMC10884920

[22] Chen T Liu WB Ren X Li YF Li W Hang CH. Whole body vibration attenuates brain damage and neuroinflammation following experimental traumatic brain injury. Front Cell Dev Biol. (2022) 10:723. doi: 10.3389/FCELL.2022.847859/BIBTEX PMC9022659 35465331

[23] Lu J Xu G Wang Y. Effects of whole body vibration training on people with chronic stroke: a systematic review and meta-analysis. Top Stroke Rehabil. (2015) 22:161–8. doi: 10.1179/1074935714Z.0000000005 26084320

[24] Steinman L. Elaborate interactions between the immune and nervous systems. Nat Immunol. (2004) 5:575–81. doi: 10.1038/NI1078 15164017

[25] Boyd RJ Avramopoulos D Jantzie LL McCallion AS. Neuroinflammation represents a common theme amongst genetic and environmental risk factors for Alzheimer and Parkinson diseases. J Neuroinflammation. (2022) 19:1–20. doi: 10.1186/S12974-022-02584-X 36076238 PMC9452283

[26] Amor S Puentes F Baker D Van Der Valk P. Inflammation in neurodegenerative diseases. Immunology. (2010) 129:154. doi: 10.1111/J.1365-2567.2009.03225.X 20561356 PMC2814458

[27] Rankin LC Artis D. Beyond host defense: emerging functions of the immune system in regulating complex tissue physiology. Cell. (2018) 173:554–67. doi: 10.1016/J.CELL.2018.03.013 29677509

[28] Yu JC Hale VL Khodadadi H Baban B. Whole body vibration-induced omental macrophage polarization and fecal microbiome modification in a murine model. Int J Mol Sci. (2019) 20:3125. doi: 10.3390/IJMS20133125 31247969 PMC6651746

[29] Nowak A Pawlak M Brychcy M Celichowski J Krutki P. Effects of brief whole-body vibration on bone metabolic and immunological indices in rats. Stud Phys Cult Tour. (2012) 19:73–6.

[30] Pawlak M Kaczmarek D Nowak A Krutki P. Low-volume whole-body vibration lasting 3 or 6 months does not affect biomarkers in blood serum of rats. Acta Physiol Hung. (2013) 100:48–53. doi: 10.1556/APHYSIOL.99.2012.003 23232704

[31] Song N Liu X Feng Q Xu M Lan X Li M. Whole body vibration triggers a change in the mutual shaping state of intestinal microbiota and body’s immunity. Front Bioeng Biotechnol. (2019) 7:377. doi: 10.3389/FBIOE.2019.00377/FULL 31850333 PMC6895539

[32] Ivanov II Tuganbaev T Skelly AN Honda K. T cell responses to the microbiota. Annu Rev Immunol. (2022) 40:559–87. doi: 10.1146/ANNUREV-IMMUNOL-101320-011829 35113732 PMC9296687

[33] Zafar H Alghadir A Anwer S Al-Eisa E. Therapeutic effects of whole-body vibration training in knee osteoarthritis: a systematic review and meta-analysis. Arch Phys Med Rehabil. (2015) 96:1525–32. doi: 10.1016/J.APMR.2015.03.010 25827655

[34] Qiu CG Chui CS Chow SKH Cheung W-H Wong RMY. Effects of whole-body vibration therapy on knee osteoarthritis: a systematic review and meta-analysis of randomized controlled trials. J Rehabil Med. (2022) 54:jrm00266. doi: 10.2340/JRM.V54.2032 35174868 PMC8963427

[35] Sakkas LI Platsoucas CD. The role of T cells in the pathogenesis of osteoarthritis. Arthritis Rheum. (2007) 56:409–24. doi: 10.1002/ART.22369 17265476

[36] Moreira-Marconi E Teixeira-Silva Y De MAG De S M-OME ACG S Reis-Silva A. Inflammatory biomarker responses to whole-body vibration in subjects with different clinical status: a systematic review. Int J Environ Res Public Health. (2022) 19:14853. doi: 10.3390/IJERPH192214853 36429572 PMC9690844

[37] Tossige-Gomes R Avelar NCP Simão AP Neves CDC Brito-Melo GEA Coimbra CC. Whole-body vibration decreases the proliferativeb response of TCD4(+) cells in elderly individuals with knee osteoarthritis. Braz J Med Biol Res. (2012) 45:1262–8. doi: 10.1590/S0100-879X2012007500139 22948377 PMC3854226

[38] Rodriguez-Miguelez P Fernandez-Gonzalo R Collado PS Almar M Martinez-Florez S de Paz JA. Whole-body vibration improves the anti-inflammatory status in elderly subjects through toll-like receptor 2 and 4 signaling pathways. Mech Ageing Dev. (2015) 150:12–9. doi: 10.1016/J.MAD.2015.08.002 26253933

[39] Ma Y He M Qiang L. Exercise therapy downregulates the overexpression of TLR4, TLR2, MyD88 and NF-κB after cerebral ischemia in rats. Int J Mol Sci. (2013) 14:3718–33. doi: 10.3390/IJMS14023718 23434667 PMC3588067

[40] Jawed Y Beli E March K Kaleth A Loghmani MT. Whole-body vibration training increases stem/progenitor cell circulation levels and may attenuate inflammation. Mil Med. (2020) 185:404–12. doi: 10.1093/MILMED/USZ247 32074302

[41] Roschel H Barroso R Batista M Ugrinowitsch C Tricoli V Arsati F. Do whole-body vibration exercise and resistance exercise modify concentrations of salivary cortisol and immunoglobulin A? Braz J Med Biol Res. (2011) 44:592–7. doi: 10.1590/S0100-879X2011007500059 21584438

[42] Pel JJM Bagheri J van Dam LM van den Berg-Emons HJG Horemans HLD Stam HJ. Platform accelerations of three different whole-body vibration devices and the transmission of vertical vibrations to the lower limbs (2009) 31:937–44. doi: 10.1016/J.MEDENGPHY.2009.05.005, 19523867

[43] Keijser JN van Heuvelen MJG Nyakas C Tóth K Schoemaker RG Zeinstra E. Whole body vibration improves attention and motor performance in mice depending on the duration of the whole-body vibration session. Afr J Tradit Complement Altern Med. (2017) 14:128–34. doi: 10.21010/ajtcam.v14i4.15 PMC5471459 28638875

[44] Boerema AS Heesterbeek M Boersma SA Schoemaker R de Vries EFJ van Heuvelen MJG. Beneficial effects of whole body vibration on brain functions in mice and humans. Dose Response. (2018) 16:1559325818811756. doi: 10.1177/1559325818811756 30574028 PMC6299320

[45] Cognitive health and older adults | National Institute on Aging. https://www.nia.nih.gov/health/cognitive-health-and-older-adults (Accessed July 7, 2023).

[46] Gocmez SS Gacar N Utkan T Gacar G Scarpace PJ Tumer N. Protective effects of resveratrol on aging-induced cognitive impairment in rats. Neurobiol Learn Mem. (2016) 131:131–6. doi: 10.1016/J.NLM.2016.03.022 27040098

[47] Gallagher M Burwell R Burchinal M. Severity of spatial learning impairment in aging: development of a learning index for performance in the Morris water maze. Behav Neurosci. (2015) 129:540–8. doi: 10.1037/BNE0000080 26214219 PMC5640430

[48] Altun M Bergman E Edström E Johnson H Ulfhake B. Behavioral impairments of the aging rat. Physiol Behav. (2007) 92:911–23. doi: 10.1016/J.PHYSBEH.2007.06.017 17675121

[49] Küçük A Gölgeli A Saraymen R Koç N. Effects of age and anxiety on learning and memory. Behav Brain Res. (2008) 195:147–52. doi: 10.1016/J.BBR.2008.05.023 18585406

[50] Cho SH Kim JH Song W. In vivo rodent models of skeletal muscle adaptation to decreased use. Endocrinol Metab. (2016) 31:31–7. doi: 10.3803/ENM.2016.31.1.31 PMC4803558 26996420

[51] Tóth K Oroszi T Nyakas C van der Zee EA Schoemaker RG. Whole-body vibration as a passive alternative to exercise after myocardial damage in middle-aged female rats: effects on the heart, the brain, and behavior. Front Aging Neurosci. (2023) 15:1034474. doi: 10.3389/FNAGI.2023.1034474 36960421 PMC10028093

[52] Regterschot GRH Van Heuvelen MJG Zeinstra EB Fuermaier ABM Tucha L Koerts J. Whole body vibration improves cognition in healthy young adults. PLoS One. (2014) 9:e100506. doi: 10.1371/JOURNAL.PONE.0100506 24949870 PMC4065066

[53] Fuermaier ABM Tucha L Koerts J Van Heuvelen MJG Van Der Zee EA Lange KW. Good vibrations – effects of whole body vibration on attention in healthy individuals and individuals with ADHD. PLoS One. (2014) 9:e90747. doi: 10.1371/JOURNAL.PONE.0090747 24587412 PMC3938804

[54] Den Heijer AE Groen Y Fuermaier ABM Van Heuvelen MJG Van Der Zee EA Tucha L. Acute effects of whole body vibration on inhibition in healthy children. PLoS One. (2015) 10:e0140665. doi: 10.1371/JOURNAL.PONE.0140665 26524188 PMC4629895

[55] Arauz YLA van der Zee EA Kamsma YPT van Heuvelen MJG. Short-term effects of side-alternating whole-body vibration on cognitive function of young adults. PLoS One. (2023) 18:e0280063. doi: 10.1371/JOURNAL.PONE.0280063 36634088 PMC9836316

[56] Amonette WE Boyle M Psarakis MB Barker J Dupler TL Ott SD. Neurocognitive responses to a single session of static squats with whole body vibration. J Strength Cond Res. (2015) 29:96–100. doi: 10.1519/JSC.0B013E31829B26CE 25536489

[57] de Bruin ED Baur H Brülhart Y Luijckx E Hinrichs T Rogan S. Combining stochastic resonance vibration with exergaming for motor-cognitive training in long-term care; a sham-control randomized controlled pilot trial. Front Med. (2020) 7:507155. doi: 10.3389/FMED.2020.507155 PMC7734185 33330519

[58] Rosado H Bravo J Raimundo A Carvalho J Marmeleira J Pereira C. Effects of two 24-week multimodal exercise programs on reaction time, mobility, and dual-task performance in community-dwelling older adults at risk of falling: a randomized controlled trial. BMC Public Health. (2021) 21:1–11. doi: 10.1186/S12889-021-10448-X/TABLES/3 34758759 PMC8582089

[59] Santin-Medeiros F Santos-Lozano A Cristi-Montero C Garatachea VN. Effect of 8 months of whole-body vibration training on quality of life in elderly women. Res Sports Med. (2017) 25:101–7. doi: 10.1080/15438627.2016.1258638 27885859

[60] Wen J Leng L Hu M Hou X Huang J. Effects of whole-body vibration training on cognitive function: a systematic review. Front Hum Neurosci. (2023) 17:854515. doi: 10.3389/FNHUM.2023.854515/BIBTEX 36845880 PMC9947405

[61] Shantakumari N Ahmed M. Whole body vibration therapy and cognitive functions: a systematic review. AIMS Neurosci. (2023) 10:130. doi: 10.3934/NEUROSCIENCE.2023010 37426779 PMC10323263

[62] Fereydounnia S Shadmehr A. Efficacy of whole body vibration on neurocognitive parameters in women with and without lumbar hyper-lordosis. J Bodyw Mov Ther. (2020) 24:182–9. doi: 10.1016/J.JBMT.2019.05.030 31987541

[63] Durgut E Orengul AC Algun ZC. Comparison of the effects of treadmill and vibration training in children with attention deficit hyperactivity disorder: a randomized controlled trial. NeuroRehabilitation. (2020) 47:121–31. doi: 10.3233/NRE-203040 32741784

[64] Yang F Estrada EF Sanchez MC. Vibration training improves disability status in multiple sclerosis: a pretest-posttest pilot study. J Neurol Sci. (2016) 369:96–101. doi: 10.1016/J.JNS.2016.08.013 27653872

[65] Kim KH Lee HB. The effects of whole body vibration exercise intervention on electroencephalogram activation and cognitive function in women with senile dementia. J Exerc Rehabil. (2018) 14:586–91. doi: 10.12965/JER.1836230.115 30276178 PMC6165986

[66] Uhm Y-H Yang D-J. The effects of whole body vibration combined computerized postural control training on the lower extremity muscle activity and cerebral cortex activity in stroke patients. J Phys Ther Sci. (2018) 30:300–3. doi: 10.1589/JPTS.30.300 29545700 PMC5851369

[67] Lam FMH Liao LR Kwok TCY Pang MYC. Effects of adding whole-body vibration to routine day activity program on physical functioning in elderly with mild or moderate dementia: a randomized controlled trial. Int J Geriatr Psychiatry. (2018) 33:21–30. doi: 10.1002/GPS.4662 28094873

[68] Aggarwal S Mortensen OV. Overview of monoamine transporters. Curr Protoc Pharmacol. (2017) 79:12.16.1. doi: 10.1002/CPPH.32 PMC5841473 29261228

[69] Ariizumi M Okada A. Effects of whole body vibration on biogenic amines in rat brain. Br J Ind Med. (1985) 42:133–6. doi: 10.1136/OEM.42.2.133 3970872 PMC1007436

[70] Ariizumi M Okada A. Effect of whole body vibration on the rat brain content of serotonin and plasma corticosterone. Eur J Appl Physiol Occup Physiol. (1983) 52:15–9. doi: 10.1007/BF00429019 6197301

[71] Okada A Ariizumi M Okamoto G. Changes in cerebral norepinephrine induced by vibration or noise stress. Eur J Appl Physiol Occup Physiol. (1983) 52:94–7. doi: 10.1007/BF00429032 6686136

[72] Yamaguchi Y. The response of monoamines in the rat brain to local vibration exposure. Sangyo Igaku. (1985) 27:73–82. doi: 10.1539/JOH1959.27.73 4068335

[73] Nakamura H Moroji T Nohara S Nakamura H Okada A. Effects of whole-body vibration stress on substance P- and neurotensin-like immunoreactivity in the rat brain. Environ Res. (1990) 52:155–63. doi: 10.1016/S0013-9351(05)80250-6 1697534

[74] Tropnikova GK. Vliianie vestibuliarnykh iader prodolovatogo mozga na funktsional’noe sostoianie serotoninergicheskoǐ sistemy mozga i tonkoǐ kishki u krys. Fiziol Zh SSSR Im I M Sechenova. (1984) 70:284–90. 6202562

[75] Jones GC Small CA Otteson DZ Hafen CW Breinholt JT Flora PD. Whole-body vibration prevents neuronal, neurochemical, and behavioral effects of morphine withdrawal in a rat model. Int J Mol Sci. (2023) 24:14147. doi: 10.3390/IJMS241814147 37762450 PMC10532581

[76] Dunnett SB Fibiger HC. Chapter 49: role of forebrain cholinergic systems in learning and memory: relevance to the cognitive deficits of aging and Alzheimer’s dementia. Prog Brain Res. (1993) 98:413–20. doi: 10.1016/S0079-6123(08)62425-5 8248529

[77] Heesterbeek M Jentsch MC Roemers P Keijser JN. Whole body vibration enhances choline acetyltransferase-immunoreactivity in cortex and amygdale neuroprotective signaling and neuroinflammation view project understanding major depressive disorder view project. J Neurol Transl Neurosci. (2017).

[78] Cariati I Bonanni R Pallone G Annino G Tancredi V D’Arcangelo G. Modulation of synaptic plasticity by vibratory training in young and old mice. Brain Sci. (2021) 11:82. doi: 10.3390/BRAINSCI11010082 33435131 PMC7827198

[79] DiSabato DJ Quan N Godbout JP. Neuroinflammation: the devil is in the details. J Neurochem. (2016) 139:136. doi: 10.1111/JNC.13607 26990767 PMC5025335

[80] Ransohoff RM Schafer D Vincent A Blachère NE Bar-Or A. Neuroinflammation: ways in which the immune system affects the brain. Neurotherapeutics. (2015) 12:896. doi: 10.1007/S13311-015-0385-3 26306439 PMC4604183

[81] Zhang W Xiao D Mao Q Xia H. Role of neuroinflammation in neurodegeneration development. Signal Transduct Target Ther. (2023) 8:267. doi: 10.1038/S41392-023-01486-5 37433768 PMC10336149

[82] Chen WW Zhang X Huang WJ. Role of neuroinflammation in neurodegenerative diseases (review). Mol Med Rep. (2016) 13:3391–6. doi: 10.3892/MMR.2016.4948 26935478 PMC4805095

[83] Shao F Wang X Wu H Wu Q Zhang J. Microglia and Neuroinflammation: crucial pathological mechanisms in traumatic brain injury-induced neurodegeneration. Front Aging Neurosci. (2022) 14:825086. doi: 10.3389/FNAGI.2022.825086 35401152 PMC8990307

[84] Smith JA Das A Ray SK Banik NL. Role of pro-inflammatory cytokines released from microglia in neurodegenerative diseases. Brain Res Bull. (2012) 87:10–20. doi: 10.1016/J.BRAINRESBULL.2011.10.004 22024597 PMC9827422

[85] Xu L Wang J Ding Y Wang L Zhu YJ. Current knowledge of microglia in traumatic spinal cord injury. Front Neurol. (2022) 12:796704. doi: 10.3389/FNEUR.2021.796704/BIBTEX 35087472 PMC8787368

[86] Schaufler D Manthou ME Theotokis P Rink-Notzon S Angelov DN. Effects of whole-body vibration and manually assisted locomotor therapy on neurotrophin-3 expression and microglia/macrophage mobilization following thoracic spinal cord injury in rats. Curr Issues Mol Biol. (2023) 45:3238–54. doi: 10.3390/CIMB45040211 37185735 PMC10137282

[87] Wang Y Ding Y Zhang W Sheng Y Chen T Wang Y. Whole-body vibration protects against early brain injury and neuroinflammation after experimental subarachnoid hemorrhage (2022) [Preprint]. doi: 10.21203/RS.3.RS-2340178/V1

[88] Oroszi T de Boer SF Nyakas C Schoemaker RG van der Zee EA. Chronic whole body vibration ameliorates hippocampal neuroinflammation, anxiety-like behavior, memory functions and motor performance in aged male rats dose dependently. Sci Rep. (2022) 12:1–10. doi: 10.1038/s41598-022-13178-1 35637277 PMC9151803

[89] Lian L Zhang Y Liu L Yang L Cai Y Zhang J. Neuroinflammation in ischemic stroke: focus on MicroRNA-mediated polarization of microglia. Front Mol Neurosci. (2021) 13:242. doi: 10.3389/FNMOL.2020.612439/BIBTEX PMC7817943 33488360

[90] Yenari MA Kauppinen TM Swanson RA. Microglial activation in stroke: therapeutic targets. Neurotherapeutics. (2010) 7:378–91. doi: 10.1016/J.NURT.2010.07.005 20880502 PMC5084300

[91] Tobin MK Bonds JA Minshall RD Pelligrino DA Testai FD Lazarov O. Neurogenesis and inflammation after ischemic stroke: what is known and where we go from here. J Cereb Blood Flow Metab. (2014) 34:1573–84. doi: 10.1038/jcbfm.2014.130 25074747 PMC4269726

[92] Raval AP Schatz M Bhattacharya P D’adesky N Rundek T Dietrich WD. Whole body vibration therapy after ischemia reduces brain damage in reproductively senescent female rats. Int J Mol Sci. (2018) 19:2749. doi: 10.3390/ijms19092749 30217051 PMC6164360

[93] Sollberger G Strittmatter GE Garstkiewicz M Sand J Beer HD. Caspase-1: the inflammasome and beyond. Innate Immun. (2014) 20:115–25. doi: 10.1177/1753425913484374 23676582

[94] Venegas C Kumar S Franklin BS Dierkes T Brinkschulte R Tejera D. Microglia-derived ASC specks cross-seed amyloid-β in Alzheimer’s disease. Nature. (2017) 552:355–61. doi: 10.1038/NATURE25158 29293211

[95] Kerr N Sanchez J Moreno WJ Furones-Alonso OE Dietrich WD Bramlett HM. Post-stroke low-frequency whole-body vibration improves cognition in middle-aged rats of both sexes. Front Aging Neurosci. (2022) 14:942717. doi: 10.3389/FNAGI.2022.942717 36062148 PMC9428155

[96] Troubat R Barone P Leman S Desmidt T Cressant A Atanasova B. Neuroinflammation and depression: a review. Eur J Neurosci. (2021) 53:151–71. doi: 10.1111/EJN.14720 32150310

[97] Dahlin E Andersson M Thorén A Hanse E Seth H. Effects of physical exercise and stress on hippocampal CA1 and dentate gyrus synaptic transmission and long-term potentiation in adolescent and adult Wistar rats. Neuroscience. (2019) 408:22–30. doi: 10.1016/J.NEUROSCIENCE.2019.03.046 30926550

[98] Ghalandari-Shamami M Nourizade S Yousefi B Vafaei AA Pakdel R Rashidy-Pour A. Beneficial effects of physical activity and crocin against adolescent stress induced anxiety or depressive-like symptoms and dendritic morphology remodeling in prefrontal cortex in adult male rats. Neurochem Res. (2019) 44:917–29. doi: 10.1007/S11064-019-02727-2/FIGURES/7 30656594

[99] Wu T Huang Y Gong Y Xu Y Lu J Sheng H. Treadmill exercise ameliorates depression-like behavior in the rats with prenatal dexamethasone exposure: the role of hippocampal mitochondria. Front Neurosci. (2019) 13:264. doi: 10.3389/FNINS.2019.00264/BIBTEX 30971882 PMC6443890

[100] Wunram HL Hamacher S Hellmich M Volk M Jänicke F Reinhard F. Whole body vibration added to treatment as usual is effective in adolescents with depression: a partly randomized, three-armed clinical trial in inpatients. Eur Child Adolesc Psychiatry. (2018) 27:645–62. doi: 10.1007/S00787-017-1071-2/FIGURES/4 29119301

[101] Peng G Yang L Wu CYCY Zhang LML Wu CYCY Li F. Whole body vibration training improves depression-like behaviors in a rat chronic restraint stress model (2021) 142:104926. doi: 10.1016/J.NEUINT.2020.104926, 33276022

[102] Jinawong K Apaijai N Chattipakorn N Chattipakorn SC. Cognitive impairment in myocardial infarction and heart failure. Acta Physiol. (2021) 232:e13642. doi: 10.1111/APHA.13642 33656800

[103] Sun W Wang Y Zheng Y Quan N. The emerging role of Sestrin2 in cell metabolism, and cardiovascular and age-related diseases. Aging Dis. (2020) 11:154. doi: 10.14336/AD.2019.0320 32010489 PMC6961765

[104] Quan N Wang L Chen X Luckett C Cates C Rousselle T. Sestrin2 prevents age-related intolerance to post myocardial infarction via AMPK/PGC-1α pathway. J Mol Cell Cardiol. (2018) 115:170–8. doi: 10.1016/J.YJMCC.2018.01.005 29325933 PMC5820139

[105] Li L Xiao L Hou Y He Q Zhu J Li Y. Sestrin2 silencing exacerbates cerebral ischemia/reperfusion injury by decreasing mitochondrial biogenesis through the AMPK/PGC-1α pathway in rats. Sci Rep. (2016) 6:1–11. doi: 10.1038/srep30272 27453548 PMC4958997

[106] Feng L Li B Cai M Zhang Z Zhao Y Yong SS. Resistance exercise alleviates the prefrontal lobe injury and dysfunction by activating SESN2/AMPK/PGC-1α signaling pathway and inhibiting oxidative stress and inflammation in mice with myocardial infarction. Exp Neurol. (2023) 370:114559. doi: 10.1016/J.EXPNEUROL.2023.114559 37788754

[107] Daixing Z Chengye Z Qiang Z Shusheng L. Upregulation of sestrin-2 expression via P53 protects against 1-methyl-4-phenylpyridinium (MPP+) neurotoxicity. J Mol Neurosci. (2013) 51:967–75. doi: 10.1007/S12031-013-0081-X/FIGURES/7 23959424

[108] Hou Y-S Guan J-J Xu H-D Wu F Sheng R Qin Z-H. Sestrin2 protects dopaminergic cells against rotenone toxicity through AMPK-dependent autophagy activation. Mol Cell Biol. (2015) 35:2740–51. doi: 10.1128/MCB.00285-15 26031332 PMC4508325

[109] Chen YS Der CS Wu CL Huang SS Yang DI. Induction of sestrin2 as an endogenous protective mechanism against amyloid beta-peptide neurotoxicity in primary cortical culture. Exp Neurol. (2014) 253:63–71. doi: 10.1016/J.EXPNEUROL.2013.12.009 24368194

[110] Skaper SD. Neurotrophic factors: an overview. Methods Mol Biol. (2018) 1727:1–17. doi: 10.1007/978-1-4939-7571-6_1 29222769

[111] Johnson EM Tuszynski MH. Neurotrophic factors In: Kordower JH Tuszynski MH, editors. CNS regeneration: basic science and clinical advances. Elsevier, Amsterdam, Netherlands: Academic Press (2008). 95–144.

[112] Åberg ND Brywe KG Isgaard J. Aspects of growth hormone and insulin-like growth factor-I related to neuroprotection, regeneration, and functional plasticity in the adult brain. Sci World J. (2006) 6:53. doi: 10.1100/TSW.2006.22 PMC5917186 16432628

[113] Wu H Zhang Y Yang X Li X Shao Z Zhou Z. Whole body vibration retards progression of atherosclerosis via insulin-like growth factor 1 in apolipoprotein E-deficient mice. Biomed Res Int. (2018) 2018:4934861. doi: 10.1155/2018/4934861 29707570 PMC5863334

[114] Li B Feng L Wu X Cai M Yu JJ Tian Z. Effects of different modes of exercise on skeletal muscle mass and function and IGF-1 signaling during early aging in mice. J Exp Biol. (2022) 225:jeb244650. doi: 10.1242/JEB.244650/277152/AM/EFFECTS-OF-DIFFERENT-MODES-OF-EXERCISE-ON-SKELETAL 36205111

[115] Oroszi T Oberman K Nyakas C van Leeuwen B van der Zee EA de Boer SF. Whole body vibration, an alternative for exercise to improve recovery from surgery? Brain Behav Immun Health. (2022) 26:100521. doi: 10.1016/J.BBIH.2022.100521 36203743 PMC9531049

[116] Lafenêtre P Leske O Ma-Högemeie Z Haghikia A Bichler Z Wahle P. Exercise can rescue recognition memory impairment in a model with reduced adult hippocampal neurogenesis. Front Behav Neurosci. (2010) 3:790. doi: 10.3389/NEURO.08.034.2009/BIBTEX PMC2831627 20204139

[117] Zhu W Fan Y Hao Q Shen F Hashimoto T Yang GY. Postischemic IGF-1 gene transfer promotes neurovascular regeneration after experimental stroke. J Cereb Blood Flow Metab. (2009) 29:1528–37. doi: 10.1038/JCBFM.2009.75 19513085 PMC2763573

[118] Hromadkova L Bezdekova D Pala J Schedin-Weiss S Tjernberg LO Hoschl C. Brain-derived neurotrophic factor (BDNF) promotes molecular polarization and differentiation of immature neuroblastoma cells into definitive neurons. Biochim Biophys Acta Mol Cell Res. (2020) 1867:118737. doi: 10.1016/J.BBAMCR.2020.118737 32389647

[119] Baydyuk M Xu B. BDNF signaling and survival of striatal neurons. Front Cell Neurosci. (2014) 8:254. doi: 10.3389/FNCEL.2014.00254 25221473 PMC4147651

[120] Park H Poo M. Neurotrophin regulation of neural circuit development and function. Nat Rev Neurosci. (2013) 14:7–23. doi: 10.1038/nrn3379 23254191

[121] Bramham C Kuipers SD Bramham CR. Brain-derived neurotrophic factor mechanisms and function in adult synaptic plasticity: new insights and implications for therapy. Curr Opin Drug Discov Devel. (2006):9, 580–6. 17002218

[122] Pesce M La FI Paolucci T Grilli A Patruno A Agostini F. From exercise to cognitive performance: role of Irisin. Appl Sci. (2021) 11:7120. doi: 10.3390/APP11157120

[123] Greenberg ME Xu B Lu B Hempstead BL. New insights in the biology of BDNF synthesis and release: implications in CNS function. J Neurosci. (2009) 29:12764–7. doi: 10.1523/JNEUROSCI.3566-09.2009 19828787 PMC3091387

[124] Zhao L He LX Huang SN Gong LJ Li L Lv YY. Protection of dopamine neurons by vibration training and up-regulation of brain-derived neurotrophic factor in a MPTP mouse model of Parkinson’s disease. Physiol Res. (2014) 63:649–57. doi: 10.33549/PHYSIOLRES.932743 24908088

[125] Cariati I Bonanni R Pallone G Romagnoli C Rinaldi AM Annino G. Whole body vibration improves brain and musculoskeletal health by modulating the expression of tissue-specific markers: FNDC5 as a key regulator of vibration adaptations. Int J Mol Sci. (2022) 23:10388. doi: 10.3390/IJMS231810388/S1 36142305 PMC9498983

[126] Cardinale M Bosco C. The use of vibration as an exercise intervention. Exerc Sport Sci Rev. (2003) 31:3–7. doi: 10.1097/00003677-200301000-00002 12562163

[127] Carstanjen B Balali M Gajewski Z Furmanczyk K Bondzio A Remy B. Short-term whole body vibration exercise in adult healthy horses. Pol J Vet Sci. (2013) 16:403–5. doi: 10.2478/PJVS-2013-0057 23971214

[128] Di Loreto C Ranchelli A Lucidi P Murdolo G Parlanti N De Cicco A. Effects of whole-body vibration exercise on the endocrine system of healthy men. J Endocrinol Investig. (2004) 27:323–7. doi: 10.1007/BF03351056 15233550

[129] Maddalozzo GF Iwaniec UT Turner RT Rosen CJ Widrick JJ. Whole-body vibration slows the acquisition of fat in mature female rats. Int J Obes. (2008) 32:1348–54. doi: 10.1038/IJO.2008.111 PMC2586051 18663370

[130] Rigamonti AE De Col A Tamini S Tringali G De Micheli R Abbruzzese L. GH responses to whole body vibration alone or in combination with maximal voluntary contractions in obese male adolescents. Growth Hormon IGF Res. (2018) 42–43:22–7. doi: 10.1016/J.GHIR.2018.07.004 30075349

[131] Wunram HL Oberste M Ziemendorff A Hamacher S Kapanci T Heller R. Differential effects of ergometer-cycling and whole-body-vibration training on serological BDNF and IGF-1 in the treatment of adolescent depression – is there an impact of BDNFp.Val66Met variants? Physiol Behav. (2021) 241:113596. doi: 10.1016/J.PHYSBEH.2021.113596 34536433

[132] Cardinale M Soiza RL Leiper JB Gibson A Primrose WR. Hormonal responses to a single session of wholebody vibration exercise in older individuals. Br J Sports Med. (2010) 44:284–8. doi: 10.1136/BJSM.2007.043232 18413339

[133] Ribeiro VGC Mendonça VA Souza ALC Fonseca SF Camargos ACR Lage VKS. Inflammatory biomarkers responses after acute whole body vibration in fibromyalgia. Braz J Med Biol Res. (2018) 51:e6775. doi: 10.1590/1414-431X20176775 29513791 PMC5856441

[134] Ribeiro VGC Lacerda ACR Santos JM Coelho-Oliveira AC Fonseca SF Prates ACN. Efficacy of whole-body vibration training on brain-derived neurotrophic factor, clinical and functional outcomes, and quality of life in women with fibromyalgia syndrome: a randomized controlled trial. J Healthc Eng. (2021) 2021:7593802. doi: 10.1155/2021/7593802 34900203 PMC8654532

[135] Simao AP Mendonca VA Avelar NCP Da FSF Santos JM Oliveira ACC. Whole body vibration training on muscle strength and brain-derived neurotrophic factor levels in elderly woman with knee osteoarthritis: a randomized clinical trial study. Front Physiol. (2019) 10:756. doi: 10.3389/fphys.2019.00756 31293437 PMC6603338

[136] Harness ET Astorino TA Knoblach SM Feather J. Change in neuroplasticity-related proteins in response to acute activity-based therapy in persons with spinal cord injury. Top Spinal Cord Inj Rehabil. (2014) 20:147–57. doi: 10.1310/SCI2002-147 25477737 PMC4252174

[137] Piotrowska A Gattner H Adamiak J Mętel S Czerwińska-Ledwig O Pilch W. Effect of whole-body vibration on serum levels of brain derived neurotrophic factor and cortisol in young, healthy women. Int J Environ Res Public Health. (2022) 19:16108. doi: 10.3390/IJERPH192316108 36498182 PMC9736339

[138] Grygiel-Górniak BPM. A review on irisin, a new protagonist that mediates muscle-adipose-bone-neuron connectivity. Eur Rev Med Pharmacol Sci. (2017) 21:4687–93. PMID: 29131244

[139] Greulich T Nell C Koepke J Fechtel J Franke M Schmeck B. Benefits of whole body vibration training in patients hospitalised for COPD exacerbations - a randomized clinical trial. BMC Pulm Med. (2014) 14:1–9. doi: 10.1186/1471-2466-14-60 24725369 PMC4021435

[140] Huh JY Mougios V Skraparlis A Kabasakalis A Mantzoros CS. Irisin in response to acute and chronic whole-body vibration exercise in humans. Metabolism. (2014) 63:918–21. doi: 10.1016/J.METABOL.2014.04.001 24814685

[141] Huberman MA d’Adesky ND Niazi QB Perez-Pinzon MA Bramlett HM Raval AP. Irisin-associated neuroprotective and rehabilitative strategies for stroke. NeuroMolecular Med. (2022) 24:62–73. doi: 10.1007/s12017-021-08666-y 34215971

[142] Wrann CD White JP Salogiannnis J Laznik-Bogoslavski D Wu J Ma D. Exercise induces hippocampal BDNF through a PGC-1α/FNDC5 pathway. Cell Metab. (2013) 18:649–59. doi: 10.1016/J.CMET.2013.09.008 24120943 PMC3980968

[143] Li D-J Li Y-H Yuan H-B Qu L-F Wang P. The novel exercise-induced hormone irisin protects against neuronal injury via activation of the Akt and ERK1/2 signaling pathways and contributes to the neuroprotection of physical exercise in cerebral ischemia. Metabolism. (2017) 68:31–42. doi: 10.1016/J.METABOL.2016.12.003 28183451

[144] De Sousa RAL Improta-Caria AC De F SBS. Exercise–linked Irisin: consequences on mental and cardiovascular health in type 2 diabetes. Int J Mol Sci. (2021) 22:2199. doi: 10.3390/IJMS22042199 33672171 PMC7926886

[145] Roriz-Filho JS Sá-Roriz TM Rosset I Camozzato AL Santos AC MLF C. (Pre)diabetes, brain aging, and cognition. Biochim Biophys Acta. (2009) 1792:432–43. doi: 10.1016/J.BBADIS.2008.12.003 19135149

[146] Munshi M Grande L Hayes M Ayres D Suhl E Capelson R. Cognitive dysfunction is associated with poor diabetes control in older adults. Diabetes Care. (2006) 29:1794–9. doi: 10.2337/DC06-0506 16873782 PMC1615865

[147] Jauch-Chara K Hallschmid M Gais S Schmid SM Oltmanns KM Colmorgen C. Hypoglycemia during sleep impairs consolidation of declarative memory in type 1 diabetic and healthy humans. Diabetes Care. (2007) 30:2040–5. doi: 10.2337/DC07-0067 17468346

[148] Wang K Song F Xu K Liu Z Han S Li F. Irisin attenuates neuroinflammation and prevents the memory and cognitive deterioration in streptozotocin-induced diabetic mice. Mediat Inflamm. (2019) 2019:1567179. doi: 10.1155/2019/1567179 PMC6590589 31281225

[149] dos Santos JM Taiar R Ribeiro VGC da Silva Lage VK Scheidt Figueiredo PH Costa HS. Whole-body vibration training on oxidative stress markers, Irisin levels, and body composition in women with fibromyalgia: a randomized controlled trial. Bioengineering. (2023) 10:260. doi: 10.3390/BIOENGINEERING10020260 36829754 PMC9952264

[150] Wang K Li H Wang H Wang JH Song F Sun Y. Irisin exerts neuroprotective effects on cultured neurons by regulating astrocytes. Mediat Inflamm. (2018) 2018:9070341. doi: 10.1155/2018/9070341 PMC6178172 30356412

[151] Cardoso ALBD Sá-Caputo DC Asad NR van Heuvelen MJ van der Zee EA Ribeiro-Carvalho A. Beneficial effects of whole-body vibration exercise for brain disorders in experimental studies with animal models: a systematic review. Behav Brain Res. (2022) 431:113933. doi: 10.1016/J.BBR.2022.113933 35654174

[152] Thompson HJ McCormick WC Kagan SH. Traumatic brain injury in older adults: epidemiology, outcomes, and future implications. J Am Geriatr Soc. (2006) 54:1590. doi: 10.1111/J.1532-5415.2006.00894.X 17038079 PMC2367127

[153] Herren K Schmid S Rogan S Radlinger L. Effects of stochastic resonance whole-body vibration in individuals with unilateral brain lesion: a single-blind randomized controlled trial: whole-body vibration and neuromuscular function. Rehabil Res Pract. (2018) 2018:1–11. doi: 10.1155/2018/9319258 PMC6093017 30155308

[154] Celletti C Suppa A Bianchini E Lakin S Toscano M La Torre G. Promoting post-stroke recovery through focal or whole body vibration: criticisms and prospects from a narrative review. Neurol Sci. (2020) 41:11–24. doi: 10.1007/S10072-019-04047-3 31468237

[155] Monteiro F Sotiropoulos I Carvalho Ó Sousa N Silva FS. Multi-mechanical waves against Alzheimer’s disease pathology: a systematic review. Transl Neurodegener. (2021) 10:36. doi: 10.1186/S40035-021-00256-Z 34560902 PMC8464104

[156] Heesterbeek M Van Der Zee EA Van Heuvelen MJG. Feasibility of three novel forms of passive exercise in a multisensory environment in vulnerable institutionalized older adults with dementia. J Alzheimers Dis. (2019) 70:681–90. doi: 10.3233/JAD-190309 31256137 PMC6700638

[157] Arenales Arauz YL Ahuja G Kamsma YPT Kortholt A van der Zee EA van Heuvelen MJG. Potential of whole-body vibration in Parkinson’s disease: a systematic review and Meta-analysis of human and animal studies. Biology. (2022) 11:1238. doi: 10.3390/BIOLOGY11081238 36009865 PMC9405106

[158] Zhao YG Lv W Huo HQ Wu JR Cheng WW Wang S. Meta-analysis of the effect of whole-body vibration training on the improvement of limb function in patients with Parkinson’s disease. Eur Rev Med Pharmacol Sci. (2023) 27:6985–95. doi: 10.26355/EURREV_202308_33270 37606148

[159] Oroszi T Geerts E Rajadhyaksha R Nyakas C van Heuvelen MJG van der Zee EA. Whole-body vibration ameliorates glial pathological changes in the hippocampus of hAPP transgenic mice, but does not affect plaque load. Behav Brain Funct. (2023) 19:5. doi: 10.1186/S12993-023-00208-9 36941713 PMC10026461

[160] Suk HJ Buie N Xu G Banerjee A Boyden ES Tsai LH. Vibrotactile stimulation at gamma frequency mitigates pathology related to neurodegeneration and improves motor function. Front Aging Neurosci. (2023) 15:1129510. doi: 10.3389/FNAGI.2023.1129510/BIBTEX 37273653 PMC10233036

[161] Strzalkowski NDJ Ali RA Bent LR. The firing characteristics of foot sole cutaneous mechanoreceptor afferents in response to vibration stimuli. J Neurophysiol. (2017) 118:1931–42. doi: 10.1152/JN.00647.2016 28679842 PMC5626905

[162] Blanco-Duque C Chan D Kahn MC Murdock MH Tsai LH. Audiovisual gamma stimulation for the treatment of neurodegeneration. J Intern Med. (2024) 295:146–70. doi: 10.1111/JOIM.13755 38115692 PMC10842797

[163] Manthou M Abdulla DSY Pavlov SP Jansen R Bendella H Nohroudi K. Whole body vibration (WBV) following spinal cord injury (SCI) in rats: timing of intervention (2017) 35:185–216. doi: 10.3233/RNN-160691, 28059803

[164] Rauch F Sievänen H Boonen S Cardinale M Degens H Felsenberg D. Reporting whole-body vibration intervention studies: recommendations of the International Society of Musculoskeletal and Neuronal Interactions. J Musculoskelet Neuronal Interact. (2010) 10:193–8. 20811143

