# Supplementary material for: The effects of whole-body vibration therapy on immune and brain functioning: current insights in the underlying cellular and molecular mechanisms
Source: Front Neurol. 2024 Jul 31;15:1422152. doi: 10.3389/fneur.2024.1422152 (PMC11323691; doi:10.3389/fneur.2024.1422152)
Supplement: Supplementary file 1 [file Data_Sheet_1.PDF]

Table 1: Summary of effects of WBV on various brain-related outcomes in preclinical studies.

Paper	Aim	Protocol	Organs/Cells studied	Molecular/ Cellular mechanisms affected	Findings
(66)	Investigated the effects of WBV on noradrenaline (NA), dopamine (DA), and serotonin (5-HT) in rats	Frequency :5-30Hz Amplitude : 0.04-0.50 mm Duration: 240mins	Brain	Monoamines: 5-HT Dopamine	NA decreased with increased acceleration. Effects on NA are dependent on the intensity of vibration.<br><br>DA in the whole brain was barely affected but a decrease was observed in the striatum and an increase in the cortex.<br><br>5-HT concentrations were increased in the hypothalamus and cerebellum.
(67)	Investigate the effects of WBV on the central nervous system, and changes in whole brain levels of Serotonin (5-HT) and 5-hydroxyindoleacetic acid (5-HIAA) were then determined.	Frequency :5, 20 & 30Hz Amplitude :0.04-0.50 mm Duration: 240 mins	Brain<br><br>Blood/plasma	5-HT<br><br>5-HIAA<br><br>Corticosterone	5-HT and 5-HIAA were significantly elevated at a frequency of 20 Hz<br><br>Increased plasma corticosterone
(69)	Investigate the effects of local vibration on the brain monoamines of rats.	Frequency :20-960Hz Amplitude :50m/s2 Duration: 240mins	Brain<br><br>Blood	NE<br>DA<br>5-HT<br>Dopamine-beta-hydroxylase (DBH)	NE in the whole brain decreased at a frequency of 120 Hz and an acceleration of 50 m/S2.<br><br>DA and 5-HT in the whole brain showed no changes

					Region-specific: NE decreased at 60 Hz and a significant decrease at 120 Hz in the hypothalamus. In the hippocampus, NE significantly decreased also at 120Hz.<br><br>DA decreased in the striatum at 20 Hz and a significant increase at 60 Hz in the medulla oblongata and pons.<br><br>5-HT significantly increased in the hypothalamus at 20 Hz and 120 Hz.
(68)	Investigate the effects of WBV on the central nervous system by examining changes in the levels of norepinephrine (NE) in both the whole brain and specific regions of interest in rats.	Frequency :5-30Hz Amplitude : 0.04-0.50 mm Duration: 240mins	Brain	NE	A significant decrease in whole brain NE levels was observed following exposure to a 5.0G acceleration at a frequency of 20 Hz.<br><br>Reductions in NE levels were also observed in the hypothalamus and the hippocampus.
(85)	Investigated the dose-dependent effects of a five-week-long WBV, including anxiety-related behavior, memory, motor	Frequency :30 Hz Amplitude :0.05-0.2mm	Brain<br><br>Blood	Microglial activation	5 mins: improved muscle grip<br>20 mins: reduced anxiety and improved spatial memory

	functions, and (neuro)inflammation	Duration: 5 or 20 mins<br>5 times a week for 5 weeks			Both: Reduced microglial activation
(71)	Compared the effect of acute WBV to the effects of a 52-54 days long chronic WBV	Frequency :10 Hz<br>Amplitude :1mm<br>Duration: 15 mins<br>Chronic: 52-54 days	Brain	5-HT	Acute: 5-HT increased in the hippocampus<br><br>Long term: 5-HT increased in parietal complex
(72)	Assessed the effectiveness of WBV as a preventive measure for the neuronal and behavioral effects of morphine withdrawal in a Wistar rat model.	Frequency : 80 Hz<br>Amplitude : NA<br>Duration: 15mins, twice daily for 5 days	Brain	Dopamine	δ-opioid receptors within cholinergic interneurons in the nucleus accumbens undergo intracellular relocation, moving from the cytosol to the cell membrane.
(48)	Explored the effects of WBV on the heart, the brain, and behaviour in female rats with induced myocardial infarction.	Frequency : 30 Hz<br>Amplitude : 0.05-0.2mm<br>Duration: 10 mins, 5 days a week for 5 weeks	Brain<br><br>Heart	Microglia<br>BDNF<br>Collagen	Anxiety-like behavior was reduced when 5 or 10-minute sessions were used, but not when 20 were used in 12-month-old female Wistar rats.<br><br>WBV reduced hippocampal (CA3) collagen content in the rats with myocardial infarction<br><br>In the CA1, WBV reduced microglia activation and normalized increased BDNF expression. In

					the CA3, WBV restored microglia activation but did not affect BDNF expression.
(40)	Examined the effects of chronic WBV on attention in mice.	Frequency : 30Hz Amplitude : 0.19mm Time: 5 or 30 mins Duration 5 weeks	Motor performance<br><br>Cognitive function		WBV has positive effects on novel object recognition and motor performance in CD1 mice
(41)	Investigate the effects of a 5-week WBV intervention on brain functions in mice (C57Bl/6J males, age 15 weeks)	Frequency :30Hz Amplitude :0.0537mm Duration: 10 mins a day, 5 days per week, for 5 weeks	Brain	Glucose uptake	WBV improves motor performance and reduces arousal-induced home cage activity.<br><br>No change in glucose uptake.
(74)	To determine whether WBV affects the cholinergic forebrain in mice.	Frequency :30Hz Amplitude :14-75mm Duration: 10 mins a day, 5 days per week, for 5 weeks	Forebrain	Choline acyltransferase (ChAT)	ChAT immunoreactivity increased in the amygdala and somatosensory cortex.
(160)	To determine which WBV-onset improves locomotor and bladder functions and influences synaptic plasticity beneficially.	SCI was followed by WBV starting 1, 7, 14, and 28 days after injury and continued for 12 weeks.<br><br>Frequency : 15Hz and 30Hz Amplitude : 1.5mm Duration: 5	Locomotion (musculo skeletal)<br><br>Bladder<br><br>Spinal cord	Synaptophysin<br><br>IBA1 (Ionized calcium-binding adaptor molecule 1 that binds with microglia)<br><br>GFAP (Glial fibrillary acidic protein)	WBV provided the most (positive effect) benefit to locomotor function (RHI), bladder function, and restoration of synaptic coverage when started 14 days after SCI compared to day 1, 7, or 28 days.

		sequential trials of 1 min at 15 Hz followed by 2 min at 30 Hz, (total 3 minutes)			
(18)	To investigate the effects of WBV intervention with an aged animal model on memory functions, anxiety-related behavior, and motor performance	Frequency :30Hz Amplitude :0.05-0.2mm Duration: 10 mins a day, 5 days per week, for 5 weeks	Brain Motor functioning	Memory<br><br>Anxiety<br><br>Motor-performance	decrease in their anxiety level<br><br>significantly increased rearing<br><br>grip strength and motor coordination were improved
(85)	To investigate the dose-dependent effects of WBV intervention with an aged animal model including anxiety-related behaviour, memory and motor functions, and neuroinflammation markers	Frequency :30 Hz Amplitude :<br><br>Duration: 5 or 20 mins 5 days per week, for 5 weeks	Brain<br><br>Hippocampus	Memory<br><br>Anxiety<br><br>Motor-performance<br><br>Neuroinflammation	20 min: Decreased anxiety-like behaviour and improved spatial memory.<br><br>5min: Muscle strength in the grip hanging test was significantly improved<br><br>Motor coordination was not significantly altered.<br><br>Microglia activation showed a significant decrease in the CA1 and Dentate gyrus subregions by both doses of WBV.<br><br>These effects were less pronounced in the CA3 and Hilus

					subregions, where only 5 min dose significantly affected microglial activation.
(112)	Compared the effects of active exercise to WBV, post-surgery	Frequency: 30 Hz<br>Amplitude: 0.05-0.2mm<br>Duration: 1st day post surgery, once 10 mins, and 2X10mins, for 14 days	Brain<br><br>Blood	DCX	Positive effects of both exercises on cognitive flexibility,<br><br>Memory did not get affected.<br><br>Neurogenesis was observed via DCX.
(98)	Investigate effects of WBV on CRS-induced depression rat model and underlying molecular mechanisms.	Frequency: 30 Hz<br>Amplitude: 4.5mm<br>Duration: 30 min per day, 6 days a week for 8 weeks	Brain<br>Blood	neuronal damage, synaptic proteins, glial cells, and trophic factors	WBV has the potential to improve behavior, inhibit neuron degeneration and glial cell pathology, enhance trophic factor expression, and facilitate the reduction of dendritic and synaptic proteins in response to stress.
(89)	Examine the impact of whole-body vibration on the outcome of ischemia in a female rat model with reproductive senescence.	Frequency: 40Hz<br>Amplitude: 0.03mm<br>Duration: 2X15mins/Day<br>For 30 days	Brain	Neuroinflammation<br>caspase-1, ASC and IL-1β<br>BDNF	Significant improvement in functional activity<br>Reduced inflammatory markers (caspase-1, ASC, and IL-1β) and infarct volume in the hippocampus.<br>Increased levels of BDNF and pTrkB
(128)	To explore the pathways by which	Frequency:20 Hz	Blood	BDNF	Increased

	exercise therapy reduces depressive symptoms in adolescents with depression and to what extent biomarkers are associated with treatment response.	Amplitude :2cm Duration: 2 mins each exercise with 2 mins rest, a total of 30 min 3–5 times weekly for 6 weeks		IGF-1	BDNF concentrations after 6-weeks intervention
(110)	Effect of WBV on Atherosclerosis Mice	Frequency :15 Hz, Amplitude :2mm Duration: 30 min for 12 weeks	Blood<br><br>Aorta	IGF-1<br>IGFR<br>IL-6	Serum: IGF1 increased<br><br>Aorta: IGF1 and IL-6 decreased
(111)	Investigate if WBV can promote muscle hypertrophy, exercise, and metabolic capacities, and activate IGF-1 signaling during early aging in mice.	Frequency :13Hz Amplitude :2 mm Duration: 15min, 5 days per week for 4 weeks	Gastrocnemius mass<br><br>Blood<br><br>Physical attribute	IGF-1<br>IGF-1R	Increased muscle mass, exercise capacity, protein synthesis, and metabolic enzyme activity.<br><br>Increased IGF1
(155)	To investigate the effects of longer duration (4 weeks) of low amplitude vibration training on dopaminergic neurons in MPTP-induced PD mice.	Frequency :10 or 30Hz Amplitude : 5mm Duration: 1X15mins /Day with 1 min rest between two bouts For 4 weeks, 5 times a week	Dopaminergic Neurons	BDNF	Vibration training(VT) could protect dopaminergic neurons from MPTP-induced damage.<br><br>The neuroprotection is probably by upregulation of BDNF<br><br>A longer duration of VT has a better effect on PD at the cellular and molecular levels.

(75)	To determine if different WBV protocols affect synaptic plasticity in young and old mice	A: 90Hz,3mins (5series), 1min recovery,12 weeks<br><br>B:45Hz, 3mins (5series), 1min recovery, 12 weeks<br><br>C:30Hz, 2.5mins(3 series), 2.5 min recovery, 12 weeks	Brain: Hippocampus	Synaptic plasticity	In young mice, only Low frequency with longer recovery periods showed positive effects.<br><br>High frequency and less time of recovery have detrimental effects and significant impairment of synaptic plasticity was observed.
(92)	To test the efficacy of post-tMCAO WBV therapy in improving cognitive deficits in reproductively senescent middle-aged female and male rats.	Frequency :40Hz Amplitude : NA Duration: Twice a day, 15 mins intervention with a 6-hour gap. 5 days a week, for 30 days.	Cognitive function<br><br>Motor function<br><br>Blood/serum Cortical tissue	Irisin<br><br>VEGF<br><br>Inflammatory cytokines (IL-1β, IL-6, IL-18, IFN-γ, and TNF-α)	Post-tMCAO WBV therapy significantly improves hippocampus-dependent cognition in middle-aged rats of both sexes.<br><br>Sex difference causes differences in the effects of WBV.<br><br>Post-tMCAO WBV significantly reduced circulating pro-inflammatory cytokines IL-1β, IL-6, IL-18, IFN-γ, and TNF-α<br><br>Significant increases in circulating VEGF levels in the rats of both sexes following WBV treatment

					compared to the No-WBV-treated group.<br><br>Post-tMCAO WBV increases circulating serum-derived EVs containing irisin
(83)	Examine the effects of WBV at the neuronal level after SCI, with a focus on changes in NT-3 expression and on microglia/macrophage reactions	Frequency: 15-Hz and 30 Hz<br>Amplitude: 1.5mm<br>Duration: 1 min at 15Hz and 2 min at 30Hz	Lumbar-spinal cord	NT-3	Initiating WBV on day 14 post-SCI had a significant functional recovery, coupled with decreased Iba1 and increased NT-3.
(84)	Examining the potential mechanism behind the therapeutic effects of WBV on subarachnoid hemorrhage (SAH) induced mice.	Frequency :30 Hz<br>Amplitude: NA<br>Duration: Twice a day for 20 days	Brain	GFAP<br>Iba-1<br>Caspase-3<br>IL-10, IL-18, and IL-1β	Reduced protein level of cleaved Caspase-3<br><br>Reduced the heightened expression of GFAP and Iba-1 resulting from SAH<br><br>WBV alleviated long-term neurological dysfunction in mice post-SAH.<br><br>WBV downregulated pro-inflammatory cytokines IL-6, IL-18, and IL-1β, while upregulating the anti-inflammatory cytokine IL-10.
(161)	To determine if different WBV protocols affect synaptic and muscle plasticity	A: 90Hz,3mins (5series), 1min	Brain: Hippocampus<br><br>Muscle: Quadriceps	Plasticity: Synaptic & Muscle	WBV can counteract long-term potentiation inhibition.

	in middle-aged mice	recovery,12 weeks<br><br>B:45Hz, 3mins (5series), 1min recovery, 12 weeks<br><br>C:45Hz, 2.5mins(3 series), 2.5 min recovery, 12 weeks.			High frequency and low recovery time are detrimental to synaptic and muscular plasticity<br><br>Both, protocols B and C showed a positive impact on synaptic and muscle plasticity.
(122)	i)To determine if different vibration exposure times and recovery periods can modulate FNDC5 expression.<br><br>ii) To determine WBV-mediated tissue adaptations for brain and musculoskeletal systems by studying tissue-specific markers: BDNF, myostatin, and collagen I	Program for 12 weeks, 36 sessions of WBV training Acceleration:2g Frequency: 45Hz<br>B:<br>5 vibration series of 3 min each with 1 min of recovery.<br><br>C:<br>3 vibration series of 2 min and 30 s each with 2 min and 30 s of recovery.	Brain<br><br>Musculoskeletal system	Synaptic plasticity<br>FNDC5<br>BDNF<br>Myostatin<br>Collagen I (COL I)	In young mice, WBV with protocol C showed positive effects in various ways:<br><br>Increased FNDC5 production, in association with improved structural organization of tissue and increased BDNF expression.<br><br>Muscle fibers have the largest mean diameter and least amount of interfibrillar connective tissue after vibration training.<br><br>Bine tissue had higher bone volume and trabecular thickness, and consequent lower trabecular separation.<br><br>Increased expression of FNDC5 in both

					muscle and bone.<br><br>Bone tissues from C had increased COL I expression<br><br>Their results show that WBV is a good strategy for muscle mass preservation
(20)	The impact of WBV pre-treatment on brain injury resulting from controlled cortical impact (CCI) in mice was studied.	Frequency: 30 Hz<br>Amplitude: NA<br>Duration: 2/day<br>20 days.	Brain in TBI<br><br>Microglia<br><br>Astrocytes	IL-10	Reduced TBI induced edema in the brain<br><br>Microglia and astrocytes activation<br><br>Increased IL-10<br><br>Suppressed apoptosis by caspase-1 regulation
(156)	Investigate molecular pathophysiology of AD in five-month-old transgenic human APP-J20 mice	Frequency: 30 Hz<br>Amplitude:0.05mm<br>Duration: 10 mins twice a day, 5 days per week, for 5 weeks	Brain in AD<br><br>Microglia<br><br>Astrocytes	CD68<br><br>Iba1<br><br>GFAP<br><br>B-amyloid	Early plaque load detection<br><br>Reduced GFAP expression<br><br>Increased microglia activity in J20 mice at approximately 6 months of age
(157)	Investigated the effects of WBV on brain pathology and motor functions	Frequency: 30 Hz<br>Amplitude: 2mm<br>Duration: 15 mins per day, 5 days a week, 4 weeks	Brain<br><br>Motor neurons	Tau (phosphorylated)<br>c-Fos (neuronal activity)<br><br>Histone (DNA repair)	Increased c-Fos-positive cells<br><br>Reduced hyperphosphorylated tau, neuronal loss, DNA damage, and loss of synaptic proteins
(103)	Investigated the effects of exercise and WBV in alleviating pre-frontal lobe injury	Frequency: 40 Hz<br>Amplitude: 0.016mm<br>Duration:	Brain	SESN2/AMPK-PGC-1α<br><br>Oxidative stress	SESN2/AMPK/PGC-1α mitigates oxidative stress and alleviates

		1h, 6 weeks			prefrontal lobe injury.
