# Supplementary material for: The effects of whole-body vibration therapy on immune and brain functioning: current insights in the underlying cellular and molecular mechanisms
Source: Front Neurol. 2024 Jul 31;15:1422152. doi: 10.3389/fneur.2024.1422152 (PMC11323691; doi:10.3389/fneur.2024.1422152)
Supplement: Supplementary file 2 [file Data_Sheet_2.PDF]

Table 2: Summary of effects of WBV on various brain-related outcomes in clinical studies.

(129)	The anabolic hormones in aged individuals were analyzed after a single session of WBV exercise.	Frequency : 30 Hz<br><br>Amplitude : 0.20 mm<br>Duration: 1 hour	Blood	Plasma cortisone<br>Testosterone<br>GH<br>IGF-1	Increased IGF-1 and cortisone<br><br>No effect on GH and testosterone
(97)	Effect of WBV on BDNF and IGF-1 in patients with depression	Frequency :20Hz<br>Amplitude :2cm<br>Duration:30 mins 3-5 times/week, 6weeks	Blood/serum	BDNF<br><br>IGF-1	Increased BDNF<br><br>The levels went down 8 weeks after the intervention was stopped<br><br>No effect on IGF-1
(125)	Investigated the impact of WBV exercise on the endocrine system in 10 healthy men.	Frequency :30Hz<br>Amplitude :4mm<br>Duration: 10X1 min with 1 min rest, and 5 min rest after 5 series (total 25 mins)	Blood<br>Plasma	Glucose<br>Cortisol<br>Norepinephrine<br>Testosterone<br>IGF-1	WBV slightly reduced plasma glucose levels<br><br>increased plasma norepinephrine concentrations
(131)	Investigated the effects of WBV on BDNF in fibromyalgia	Frequency : 35-40Hz<br>Amplitude : 4mm<br>Duration: 3 mins to 10.6 mins 3 times per week for 6 weeks	Blood	BDNF	Increased BDNF expression
(130)	Investigated the effects of acute WBV on inflammatory biomarkers in fibromyalgia	Frequency : 35-40Hz<br>Amplitude : 4mm<br>Duration: 1 bout	Blood	BDNF<br>sTNFR1<br>sTNFR2<br>Adiponectin<br>Leptin<br>Resistin	No significant difference between vibration and non-vibration groups
(132)	To evaluate the effects of adding	Frequency : 35-40Hz	Plasma<br>Brain	BDNF	Improved lower lib performance

	WBV to squat training on isometric quadriceps muscle strength (IQMS) and plasma levels of BDNF in elderly women with knee osteoarthritis (kOA).	Amplitude: 4mm<br>Duration: 12 weeks	Musculoskeletal		possibly due to increased BDNF
(128)	To investigate the effects of ergometer cycling and WBV in adolescents with depression	Frequency: 20Hz<br>Amplitude: 2cm<br>Duration: 30 min (2-3mins per exercise) 3–5 times weekly for 6 weeks	Blood serum	BDNF<br>IGF-1	6 weeks of both interventions increased BDNF<br><br>IGF-1 levels only increased by active exercise.<br><br>No correlation between these factors to depression
(133)	To investigate acute changes in neuroplasticity-associated proteins during Activity-Based Therapy (ABT) in individuals with spinal cord injury (SCI).	Frequency: 35 Hz -<br>Amplitude: 2 mm<br>Duration: 15, 1-minute bouts of vibration interspersed with 1 minute of rest.	Brain<br>Spinal cord<br>Serum	BDNF	No significant changes in BDNF
(134)	To assess the effect of vibration training BDNF and cortisol in young women.	Frequency: 20-60 Hz<br>Amplitude: 2 and 4 mm<br>Duration: 15, 1-minute bouts of vibration interspersed with 1 minute of rest.	Brain - Blood (serum)	BDNF<br>Cortisol	No statistically significant changes in BDNF and cortisol levels were observed
(137)	To investigate the effect of acute and	Frequency: 20-60 Hz	Blood	Irisin	Baseline (Week 0):

	chronic WBV exercise on circulating irisin levels in young healthy subjects.	Amplitude: 2.5 and 5 mm<br>Duration: 6-week program of WBV training with 2 sessions per week. Per session was 11-18.5 mins			Resting irisin levels remained consistent.<br>Acute Vibration Exercise:<br>Significantly increased circulating irisin levels by 9.5%.<br>Chronic Training (6 Weeks):<br>No change in baseline irisin levels.
(146)	Investigate the effect of WBV on oxidative stress markers, plasma irisin levels, and body composition in women with fibromyalgia.	Frequency: 35-40 Hz<br>Amplitude: 4 mm<br>Duration: 3 times a week for 6 weeks.<br>3 to 10.6 min per session	Blood<br>Plasma	Irisin<br>Oxidative stress	WBV reduced innate immune response. - Improved body composition and increased plasma irisin levels in women with FM. - The observed effects may be mediated via irisin.
