# Supplementary material for: The effects of whole-body vibration therapy on immune and brain functioning: current insights in the underlying cellular and molecular mechanisms
Source: Front Neurol. 2024 Jul 31;15:1422152. doi: 10.3389/fneur.2024.1422152 (PMC11323691; doi:10.3389/fneur.2024.1422152)
Supplement: Supplementary file 3 [file Data_Sheet_3.PDF]

## Methods

The search strategy was designed to examine the effects of WBV on molecular and cellular pathways involved in immune and brain function in humans, animals, and cellular studies. The following databases were used to collect literature: PubMed, Google Scholar, and Scopus.

We used the following key search terms for PubMed and the other databases: (“Whole Body Vibration” OR “Vibration Therapy" OR “Vibration exercise” OR “Vibration Training”) AND ("Molecular effects on the immune system" OR "Cellular effects on immune system" OR "Immune system" OR "Cytokines") OR ("Molecular effects on brain" OR “Cellular effects on brain” OR “Brain” OR "Neurotransmission" OR "Neuroprotection" OR "Neuroinflammation" OR "Brain disorders" OR "Neurodegeneration").

### Study selection

We used the Mendeley reference management tool to remove duplicate records identified by the databases. G.A. initially screened the titles and abstracts based on the eligibility criteria. Subsequently, G.A. reviewed the full texts for inclusion in the qualitative synthesis, applying the same criteria. In cases of uncertainty, the other authors (E.A.v.d.Z. and A.K.) also screened the full texts. T.O. and M. J. G. v. H suggested any other relevant papers. Any disagreements regarding article selection were resolved by mutual consensus among all co-authors. Finally, we manually searched the reference lists of each selected article and reviewed articles to identify additional relevant articles.

### Eligibility Criteria

Studies were selected if they discussed any cellular or molecular effects of WBV. Exception was made for the brain functioning section (section 2.1) where we summarized the effects of WBV on brain functioning before diving into the molecular pathways. This segment was included to contextualize the functional changes potentially driven by modifications at the molecular level which were discussed later throughout the review. We included all papers, without any age limitation but excluded those studying the detrimental effects of WBV using very long (>60 min) sessions, high (> 80 Hz) frequency, or large amplitudes. Two of the excluded studies were nevertheless mentioned in the review to highlight the importance of exploring specific pathways using less detrimental frequencies and shorter durations. Studies lacking proper protocol reporting were excluded. Only papers published in English or with a reliable English translation were included; studies in other languages were excluded.
